# Development of a CRISPR-Cas13-based antiviral strategy against hepatitis E virus

**DOI:** 10.1016/j.jhepr.2026.101885

**Published:** 2026-05-04

**Authors:** Emely Richter, Mara Klöhn, Maximilian K. Nocke, Marcel Edgar Friedrich, Daniel Todt, Eike Steinmann, Yannick Brüggemann

**Affiliations:** 1 Department of Molecular and Medical Virology, Ruhr University Bochum, Bochum, Germany; 2 Hepatitis E Virus Research Hub (HepE-Hub), Bochum, Germany; 3 Department of Translational and Computational Infection Research (TRACiR), Ruhr University Bochum, Bochum, Germany; 4 European Virus Bioinformatics Center (EVBC), Jena, Germany; 5 German Centre for Infection Research (DZIF), External Partner Site, Bochum, Germany

**Keywords:** Hepatitis E virus (HEV), CRISPR-Cas13, Antivirals

## Abstract

**Background & Aims:**

Effective antiviral drugs remain unavailable for many clinically relevant pathogens, including the hepatitis E virus (HEV). This study aimed to evaluate the CRISPR/Cas13d system as a potential antiviral strategy against HEV.

**Methods:**

We developed a reporter assay to screen CRISPR RNAs (crRNAs) targeting conserved regions of the HEV genome and tested their antiviral activity in human hepatoma cells using a robust HEV cell culture model. HEV replication was assessed using a subgenomic replicon, infectious particle production was quantified by immunofluorescence and titration assays. A bioinformatic analysis was performed to identify a minimal set of crRNAs capable of broadly targeting circulating human pathogenic HEV strains.

**Results:**

A crRNA screen identified multiple functional crRNAs targeting HEV-3, with ORF1-targeting crRNAs significantly reducing viral capsid expression (*p <*0.01) and the number of HEV-infected cells (*p <*0.01). Cas13d-mediated targeting led to robust reduction of HEV replication and markedly lowered infectious virus production *in vitro* (*p <*0.001). Bioinformatic analysis revealed that just three distinct crRNAs could cover ∼94% of known HEV genomes with zero mismatches, while four crRNAs achieved complete coverage.

**Conclusions:**

Our findings demonstrate that CRISPR/Cas13d can target HEV replication and viral progeny production *in vitro*. The identification of a minimal crRNA set capable of broadly targeting circulating HEV strains suggests that the CRISPR/Cas13d system may offer an antiviral strategy to address challenges related to viral evolution and treatment escape.

**Impact and implications:**

This study establishes CRISPR/Cas13d as a proof-of-concept antiviral strategy against hepatitis E virus (HEV), demonstrating suppression of viral replication and particle production *in vitro*. By identifying a minimal set of broadly effective crRNAs, we provide a framework for targeting diverse HEV variants and buffering against viral evolution. These findings highlight the potential of CRISPR-based systems as innovative antiviral strategies.

## Highlights

• CRISPR/Cas13d enables efficient targeting of HEV RNA sequences.
• ORF1-targeting crRNAs reduce viral replication, capsid expression, and infectious HEV production in hepatoma cells.
• A combination of three to four crRNAs provides broad coverage of circulating HEV genomes, potentially buffering viral escape mutations.

## Graphical abstract

**Figure:**
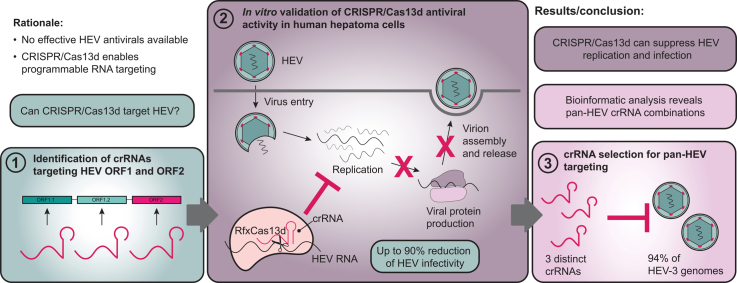

## Introduction

The hepatitis E virus (HEV, species *Paslahepevirus balayani*) is a long-overlooked RNA virus and the primary cause of acute viral hepatitis in humans globally. Each year, HEV causes an estimated 20 million infections, 3.3 to 19 million acute cases, and 3,450 to 70,000 deaths, with wide estimate ranges due to inconsistent surveillance and reporting, especially in low- and middle-income countries.^1^^,^^2^ In addition, HEV ranks sixth among viruses with a high spillover risk, underscoring its significant potential for zoonotic transmission.^3^ While HEV infections are typically self-limiting and asymptomatic in immunocompetent individuals, they can progress to chronicity in immunocompromised patients and cause fulminant hepatitis in high-risk groups, such as pregnant women.^4^^,^^5^ Current therapeutic options for HEV are limited to the off-label use of the broad-spectrum antiviral agent ribavirin (RBV).[6], [7], [8] However, RBV is contraindicated in pregnancy owing to its teratogenicity and its use is further restricted by suboptimal efficacy, poor tolerability, and a range of side effects. Moreover, HEV variants emerging in response to antiviral treatment have been identified.[9], [10], [11], [12] Although the emergence of viral variants may contribute to RBV resistance, a causal link to treatment failure has yet to be established. Collectively, these limitations underscore an urgent need for the development of novel and safer antiviral strategies.

In this context, the discovery of RNA-targeting CRISPR/Cas systems in bacteria has generated tremendous interest in antiviral research.^13^ The CRISPR/Cas13 system functions similarly to the well known CRISPR/Cas9 system. However, unlike the Cas9 endonuclease which targets DNA, the Cas13 enzyme targets and cleaves single-stranded RNA. The diverse Cas13 family contains at least four known subtypes (Cas13a, Cas13b, Cas13c, and Cas13d). Cas13 enzymes use so-called CRISPR-associated RNAs (crRNAs) that contain a customizable 22-nt spacer sequence that can direct the Cas13 protein to specific RNA molecules for targeted RNA degradation.^14^ Hence, the specific RNA endonuclease activity of the Cas13 protein can be "programmed" to specifically recognize and target transcripts or viral RNA genomes. In initial studies, Cas13 was demonstrated to mitigate enterovirus, influenza and SARS-CoV-2 infections *in vitro* in human cells and *in vivo* in rodent models.[15], [16], [17] Here we explored whether Cas13d could be used to target HEV *in vitro* in human cells in an analogous manner.

## Materials and methods

### Cell culture

HepG2 (ATCC-Nr.: HB-8065) and HEK293T cells (ATCC-Nr.: CRL-3216) cells were grown in DMEM-high-glucose (Gibco, #11965) supplemented with 10% (v/v) FCS (Capricorn, Lot. Nr. CPC21-4114), 1% (v/v) non-essential amino acids (Gibco, #11140050), 2 mM L-glutamine (Gibco, #25030), 100 IU/ml penicillin and 100 μg/ml streptomycin (Gibco, #15140) (DMEM complete). HepG2/C3A cells (ATCC-Nr.: HB-8065) were grown in Eagle's minimum essential medium (Gibco, #11095) supplemented with 10% (v/v) ultra-low IgG FCS (Gibco, #16250-078), 1% (v/v) non-essential amino acids, 2 mM L-glutamine, 100 μg/ml gentamicin (Gibco, #15710) and 1 mM sodium pyruvate (Gibco, #11360). HepG2 and HepG2/C3A cells were grown on rat collagen-coated (SERVA Electrophoresis, #47256.01) cell culture dishes (Sarstedt) and incubated at 37 °C in a 5% CO_2_ incubator.

Additional materials and methods describing assays used in this study are specified in the supplementary information.

## Results

**Fig. 1:**
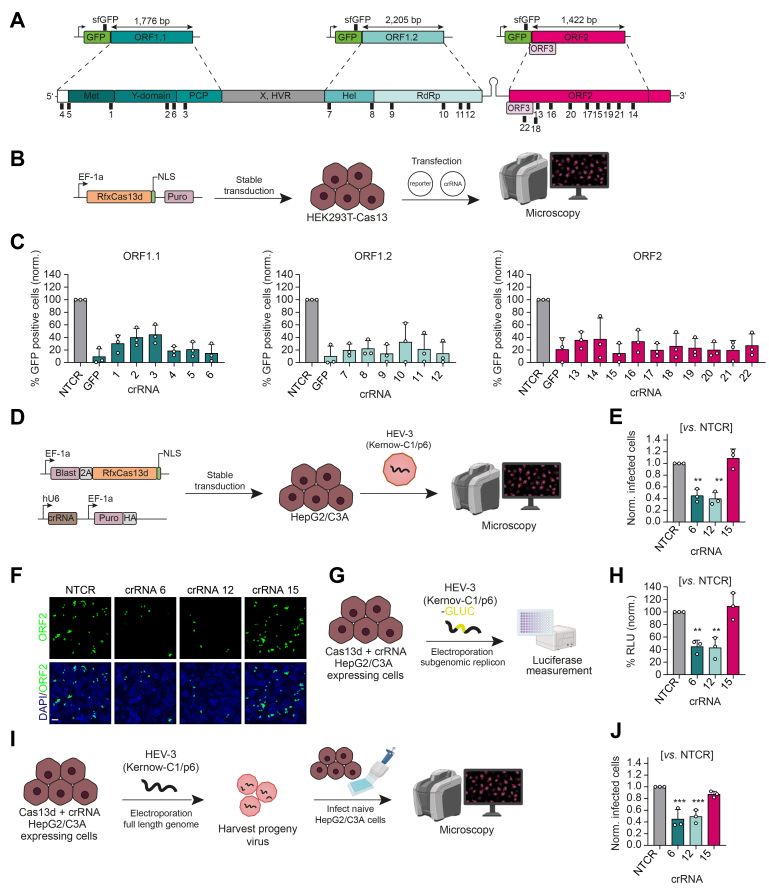
Cas13d-mediated targeting of HEV *in vitro*. (A) Schematic of HEV reporters (ORF1.1; ORF1.2 and ORF2) created with sequences of the HEV-3/p6 fused to sfGFP and selected crRNAs. (B) Experiment workflow to challenge Cas13d-NLS expressing HEK293T cells with HEV reporters and crRNAs. (C) Quantification of relative GFP expression 24 h after transfection (means + SD; n = 3). ORF1.1 (left); ORF1.2 (middle) and ORF2 (right). (D) HepG2/C3A cells stably expressing Cas13d-NLS-FLAG and a crRNA were infected with HEVp6 and stained for viral capsid protein after 3 days. (E) Quantification of relative infected cells (means + SD; n = 3). (F) Immunofluorescence images of HepG2/C3A cells 72 h after infection with HEV (Kernow-C1/p6; MOI 1). Viral capsid protein (ORF2) = green; DAPI = blue. Scale bar = 100 μm. (G) HepG2/C3A cells stably expressing Cas13d-NLS-FLAG and a crRNA were electroporated with HEV p6 GLUC RNA (Kernow-C1/p6-GLUC). GLUC signal in supernatant was quantified 3 days after electroporation. (H) RLU for each crRNA after HEV p6 GLUC electroporation normalized to the NTCR (means + SD; n = 3). (I) HepG2/C3A stably expressing Cas13d-NLS-FLAG and a crRNA were electroporated with HEV full-length RNA (Kernow-C1/p6). Virus was harvested and titrated after 7 days. The number of infected cells was quantified 7 days after titration. (J) Relative infected cells for each Cas13d/crRNA cell line after virus titration for infectious particle production assay normalized to the NTCR (means + SD; n = 3). Statistical significance in (E), (H) and (J) was determined using a one-way ANOVA with Dunnett’s post hoc test. (∗∗∗*p <*0.001 and ∗∗*p <*0.01, non-significant values are not shown). GLUC, *Gaussia luciferase*; HEV, hepatitis E virus; MOI, multiplicity of infection; NLS, nuclear localization signal; NTCR, non-targeting control RNA; ORF, open reading frame; RLU, relative light units; sfGFP, superfolder green fluorescent protein.

To identify effective and specific crRNA sequences to target and cleave HEV RNA within cells, we developed a reporter assay based on fragments of the HEV-3 *Kernow-C1/p6* genome fused to GFP (ORF1.1, ORF1.2 and ORF2). Candidate crRNAs targeting conserved regions of the HEV genome were designed using a Cas13d-specific crRNA prediction algorithm.^18^ We selected 22 crRNAs targeting conserved regions within the open reading frame 1 (ORF1) or 2 (ORF2). crRNAs with predicted off-target binding in the human transcriptome, defined as any site with ≤2 mismatches, were excluded (Fig. 1A). To test crRNA activity, we created a HEK293T cell line (HEK293T-Cas13d) stably expressing RfxCas13d-nuclear localization sequence (NLS). Reporter and crRNA expressing plasmids were transfected into HEK293T-Cas13d and GFP fluorescence was determined 24 h post-transfection (Fig. 1B). A crRNA targeting GFP was used as a positive control. We observed that all HEV-specific crRNAs reduced reporter expression compared to a non-targeting control, demonstrating that Cas13d is an effective system for targeted degradation of HEV RNA in human cells (Fig. 1C). From this initial screening we selected the most effective crRNAs (6; 12; 15) for each reporter construct to test the antiviral potential of Cas13d against HEV.

**Fig. 2:**
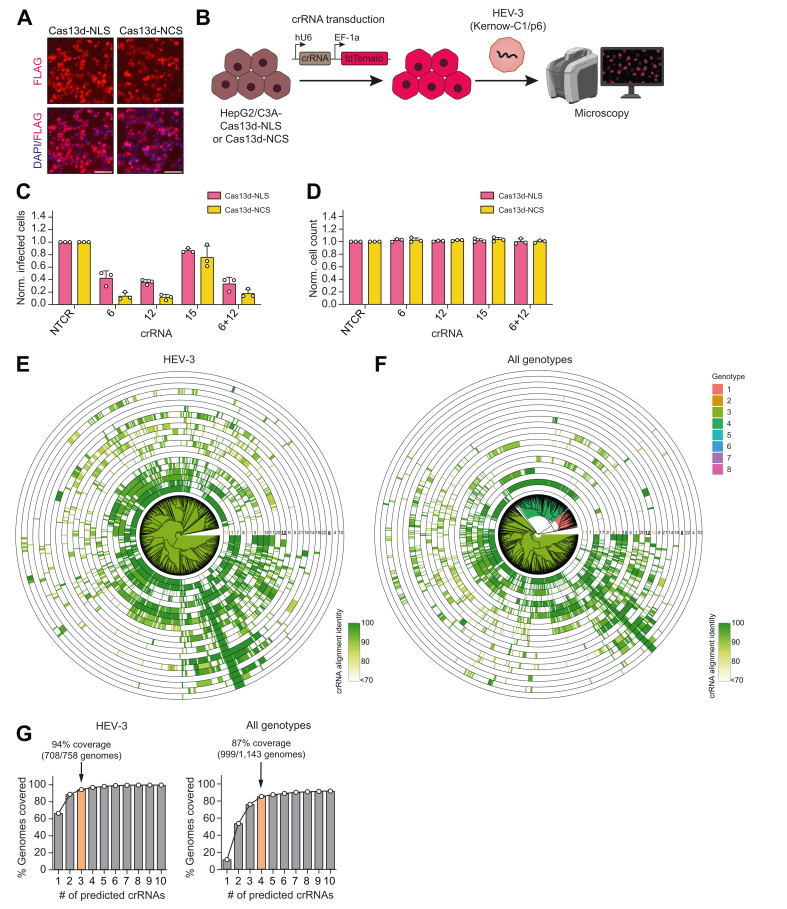
Cas13d-NCS enhances antiviral activity against HEV and pan-HEV targeting using a minimal crRNA pool. (A) Immunofluorescence images of HepG2/C3A stably expressing Cas13d-NLS-FLAG or Cas13d-NCS-FLAG. Cas13-NLS/NCS-FLAG = red; DAPI = blue. All scale bars = 100 μm. (B) Experiment workflow to challenge Cas13d expressing HEK293T cells with HEV reporters and crRNAs. (C) Quantification of HEV-infected cells and (D) relative cell number based on nuclear counts (D) in HepG2/C3A cells stably expressing Cas13d-NLS-FLAG or Cas13d-NCS-FLAG following crRNA transduction and 96 h post-infection with HEV (Kernow-C1/p6; MOI 0.1). (E and F) Pan-HEV targeting using a minimal crRNA pool. Inner ring: A phylogenetic tree of all (E) 758 HEV-3 genomes or (F) 1,143 genomes representing all HEV genotypes analyzed, organized according to subtype or genotype classification. Outer ring: Coverage of individual HEV genomes by the initially tested 21 complementary crRNAs (excluding crRNA 15), with targeted genomes indicated in green. crRNA 6 and crRNA 12 are highlighted in bold. The minimum set of crRNAs identified in (G) is indicated in orange. (G) Predicted minimum number of crRNAs required to achieve coverage of the majority of analyzed HEV-3 genomes (n = 758) or genomes representing all HEV genotypes (n = 1,143). HEV, hepatitis E virus; MOI, multiplicity of infection; NCS, nucleocytoplasmic shuttling signal; NLS, nuclear localization signal; ORF, open reading frame; NTCR, non-targeting control RNA.

We therefore generated HepG2/C3A cells stably expressing RfxCas13d-NLS (HepG2/C3A-Cas13d) along with individual crRNAs (6; 12; 15) and challenged them with HEVp6 (Figs 1D and S1A). Immunofluorescence analysis performed 72 h post-infection revealed a reduction in viral capsid (ORF2) expression in cells expressing on-target crRNAs against ORF1 (6 and 12) compared to non-targeting controls (Fig. 1E, F). In contrast, crRNA 15 targeting ORF2 had no effect on the number of infected cells. Quantification of infected cells confirmed that crRNAs against ORF1 (6 and 12) significantly decreased the number of HEV-infected cells up to ∼50% (Fig. 1E, F). Total cell numbers remained unchanged, indicating that combined Cas13/crRNA expression can reduce HEV infection in hepatoma cells without compromising cell viability, even under activation of Cas13d cleavage activity (Fig. S1B). We next examined whether Cas13d could reduce HEV replication and infectious virus production. HepG2/C3A-Cas13d cells stably expressing crRNAs were electroporated with HEV *Gaussia* luciferase RNA, and viral replication was quantified by *Gaussia* luciferase activity in the supernatant (Fig. 1G). Consistent with the infection data, crRNAs targeting ORF1 (6 and 12) caused a pronounced reduction in luciferase signal compared to non-targeting controls (Fig. 1H). In contrast, the ORF2-targeting crRNA 15 had no effect on replication. To assess the impact on infectious particle production, Cas13d/crRNA-expressing cells were electroporated with full-length HEV RNA, and progeny virus was harvested and titrated after 7 days (Fig. 1I). Immunofluorescence staining against ORF2 revealed a strong reduction in infected cells for lines expressing ORF1-targeting crRNAs 6 and 12, whereas the ORF2-targeting crRNA 15 did not reduce viral progeny production (Fig. S1C). Quantification confirmed that ORF1-targeting crRNAs substantially decreased the production of infectious HEV particles (Fig. 1J). Together, these results demonstrate that Cas13d can reduce HEV replication and the generation of infectious virus *in vitro*. However, the efficiency of the Cas13d-NLS construct in our system was only ∼50%, which motivated us to investigate alternative designs. To address this limitation, we explored a nucleocytoplasmic shuttling Cas13d (Cas13d-NCS) construct that fuses nuclear localization and export signals to enhance cytosolic RNA targeting.^19^ To assess whether Cas13d-NCS improves antiviral activity against HEV, we generated HepG2/C3A cells stably expressing either Cas13d-NLS or Cas13d-NCS (Fig. 2A), delivered crRNAs via lentiviral transduction, and subsequently infected the cells with HEV (Fig. 2B). Compared to Cas13d-NLS, Cas13d-NCS markedly reduced HEV infectivity by up to ∼90% with crRNAs targeting ORF1 (6 and 12), whereas crRNA 15 against ORF2 had no effect (Fig. 2C). Total cell numbers (Fig. 2D) and viability (Fig. S2C) were unaffected, indicating that Cas13d-NCS efficiently suppresses HEV infection without compromising cell health. Combining crRNAs 6 and 12, with half the lentivirus used per crRNA, maintained similar levels of HEV suppression (Fig. 2C), indicating that crRNAs can be effectively combined without loss of activity. Because combining individual crRNAs potentially allows for broad targeting of HEV genotypes, we next asked whether it is possible to design a minimal set of crRNAs that could effectively target the majority of known HEV genomes. Through bioinformatic analysis, we aligned 1,143 full-length HEV genomes against our initial panel of crRNAs (excluding the inefficient crRNA 15) (Fig. 1A) and iteratively refined this set to identify the smallest subset of crRNAs capable of targeting all known HEV genomes with zero mismatches (Fig. 2E, F). We found that just three crRNAs could target ∼94% (708/758) of HEV-3 genomes, and a panel of four distinct crRNAs covered ∼87% (975/1,143) across all HEV genotypes with no mismatches (Fig. 2G). The ability to use a relatively small number of crRNAs to broadly target all HEV strains and thereby also potentially buffer against viral evolution and escape highlights the unique and versatile potential of the CRISPR/Cas13 system as an antiviral strategy.

## Discussion

In this study, we repurposed the RNA-guided endonuclease activity of Cas13d to target HEV in human cells. We demonstrated that Cas13d can efficiently degrade HEV RNA, suppress viral replication, and reduce infectious particle production in hepatoma cells. The lack of antiviral activity of the ORF2-targeting crRNA may reflect limited accessibility of the target site. While active in the reporter assay, ORF2 crRNAs failed to suppress HEV replication or particle production, likely because ORF2 is translated from a subgenomic RNA expressed late in infection, rather than the full-length genomic RNA. Testing additional ORF2-targeting crRNAs could clarify whether this limitation is site-specific or reflects broader challenges in targeting the subgenomic RNA. Bioinformatic analysis further revealed that as few as three crRNAs could target ∼94% of sequenced HEV-3 genomes, and a panel of four crRNAs could target ∼87% (975/1,143) of genomes across all HEV genotypes. Unlike Cas9, Cas13d is capable of processing its own CRISPR array,^20^ enabling the encoding of multiple guides within a single construct to simultaneously and effectively suppress diverse viral variants.^21^ This ability to achieve broad strain coverage with minimal guides not only buffers against viral evolution and escape but also underscores the unique therapeutic potential of Cas13 compared to conventional antivirals.^22^ While our findings establish Cas13d as a proof-of-concept antiviral strategy against HEV *in vitro*, several challenges must be addressed before clinical translation. Importantly, our experiments tested Cas13d as a prophylactic system introduced prior to viral challenge; however, we hypothesize that it could also be effective in reducing viral load post-infection, as previously demonstrated for coronaviruses.^21^ In addition, we employed stable Cas13d/crRNA-expressing cell lines, which is not compatible with immediate translational applications. A major barrier to clinical deployment of Cas13 remains the development of safe and efficient *in vivo* delivery methods. Future efforts should integrate crRNA selection strategies with predictive algorithms to streamline guide validation, while optimizing delivery methods in relevant *in vivo* models, determining safe and effective dosing regimens, and assessing the immunogenicity of both Cas13d and its delivery vehicles.^23^ Collateral RNA cleavage, reported under specific circumstances, must also be carefully evaluated and minimized by carefully regulating RfxCas13d.^24^ As Cas13 biology advances, the discovery of more compact, efficient, and less immunogenic RNA-targeting variants could further enhance its therapeutic potential. Notably, the crRNA discovery and validation pipeline established here for HEV can be readily adapted to other CRISPR systems, such as the recently developed type III CRISPR-Csm platform,^25^ offering a generalizable framework for next-generation CRISPR-based HEV antivirals.

## Abbreviations

crRNA, CRISPR RNAs; HEV, hepatitis E virus; NCS, nucleocytoplasmic shuttling sequence; NLS, nuclear localization sequence; ORF, open reading frame; RBV, ribavirin; NTCR, non-targeting control RNA.

## Authors’ contributions

Contributed to the conception and design of the study: YB, ES. Provided administrative, study supervision, and obtained funding: YB, ES, DT. Performed experiments and substantially contributed to the acquisition of data and its analysis: ER, MKL, MN, MEF, YB. Interpretation of data: ER, MKL, MN, MEF, YB, ES, DT. Drafted the manuscript: YB, ES. Revised the manuscript critically for important intellectual content: All authors.

## Data availability

All data supporting the findings of this study are included within the article and its supplementary information. Raw data are available upon reasonable request.

## Financial support

ES was supported by the German Research Foundation (STE 1954/16-1) and German Centre for Infection Research (DZIF, TTU 05.823_00). DT was supported by the German Federal Ministry of Research, Technology and Space (BMFTR, project VirBio; 01KI2106). YB was supported by German Research Foundation (DFG, BR 7111/1-1) and the Medical Faculty -FoRUM program of the Ruhr University Bochum (F1030-2021). ER was supported by the Hannover Graduate School for Neurosciences, Infection Medicine and Veterinary Sciences (HGNI) of the University of Veterinary Medicine Hannover (TiHo).

## Conflicts of interest

Emely Richter: nothing to disclose. Mara Klöhn: nothing to disclose. Maximilian K. Nocke: nothing to disclose. Daniel Todt: nothing to disclose. Eike Steinmann: nothing to disclose. Yannick Brüggemann: nothing to disclose.

Please refer to the accompanying ICMJE disclosure forms for further details.
