# Supplementary material for: Development of a CRISPR-Cas13-based antiviral strategy against hepatitis E virus
Source: JHEP Rep. 2026 May 4;8(7):101885. doi: 10.1016/j.jhepr.2026.101885 (PMC13277442; doi:10.1016/j.jhepr.2026.101885)
Supplement: Multimedia component 1 [file mmc1.pdf]

Development of a CRISPR-Cas13-based antiviral strategy against hepatitis E virus

Emely Richter, Mara Klöhn, Maximilian K. Nocke, Marcel Edgar Friedrich, Daniel Todt, Eike Steinmann, Yannick Brüggemann

Table of contents

Supplementary materials and methods……………………………………………….2

Fig. S1………………………………………………………………………………….7

Fig. S2………………………………………………………………………………….8

Table S1………………………………………………………………………………..9

Supplementary materials and methods

Plasmids

pSLQ5079_pHR_PGK_sfGFP_CoV-F1 (Addgene plasmid #155303), pSLQ5465_pHR_hU6-crScaffold_EF1a-PuroR-T2A-BFP (Addgene plasmid #155307), pSLQ5429_pUC_hU6-crScaffold_EF1a-BFP (Addgene Plasmid #155306) and pSLQ5428_pHR_EF1a-mCherry-P2A-Rfx_Cas13d-2xNLS-3xFLAG (Addgene plasmid #155305) were a gift from Stanley Qi. pLentiRNACRISPR_005 - hU6-DR_BsmBI-EFS-RfxCas13d-NLS-2A-Puro-WPRE (Addgene plasmid plasmid #138147) was a gift from Neville Sanjana. pCMVR8.74 (Addgene plasmid # 22036) and pMD2.G (Addgene plasmid # 12259) were a gift from Didier Trono. SGL40C.EFS.dTomato (Addgene plasmid #89395) was a gift from Dirk Heckl. Plasmids encoding the full-length viral genome of the Kernow-C1/p6 virus isolate or Gaussia luciferase reporter replicon (Kernow-C1/p6 strain with a truncated ORF2 replaced with a Gaussia luciferase gene) were a gift from Sue Emerson. Plasmids were assembled using Gibson assembly (New England Biolabs, E2611L) with inserts PCR-amplified using Q5 high-fidelity DNA polymerase (NEB, M0491L) and primers containing appropriate overhangs. To generate Blast-2A-RfxCas13d-NLS, the mCherry sequence in pSLQ5428_pHR_EF1a-mCherry-P2A-Rfx_Cas13d-2xNLS-3xFLAG (Addgene plasmid #155305) was replaced with a blasticidin resistance gene. Blast-2A-RfxCas13d-NCS was generated by replacing the NLS sequence with a previously described NCS sequence consisting of NLS–NLS–NES. To generate hU6-crScaffold_EF1a-tdTomato, the Cas9 direct repeat in SGL40C.EFS.dTomato was replaced with a modified Cas13d direct repeat from pLentiRNAGuide_001 (Addgene plasmid #138150). To generate RfxCas13d-NLS-2A-Puro, the hU6-DR_BsmBI region of pLentiRNACRISPR_005 was deleted using the Q5 Site-Directed Mutagenesis Kit (NEB, E0554S). To generate pUC_hU6-crScaffold_EF1a-FLAG, the BFP region in pSLQ5429_pUC_hU6-crScaffold_EF1a-BFP was replaced with a 3x FLAG using the Q5 Site-Directed Mutagenesis Kit (NEB, E0554S). HEV sensor sequences, derived from the Kernow-C1/p6 strain, were cloned into pSLQ5079_pHR_PGK_sfGFP_CoV-F1 (Addgene plasmid #155303) by replacing the CoV sequences to generate HEV-ORF1.1, HEV-ORF1.2 or HEV-ORF2 sensors.

crRNA design and cloning

crRNA sequences were designed using the Cas13 design tool (https://cas13design.nygenome.org/. Only crRNAs predicted to have no off-target binding (over 2 mismatches per crRNA) within the human transcriptome were used. crRNA expression plasmids were generated using standard restriction–ligation cloning. In brief, forward and reverse oligonucleotides corresponding to each spacer were phosphorylated, annealed, and ligated into the backbone using T4 DNA ligase. The assembled inserts were cloned into either the pHR backbone (Addgene plasmid #155307), the modified SGL40C.EFS.dTomato

backbone or the pUC19 backbone (Addgene plasmid #155306). All spacer sequences for the crRNAs are listed in Table S1.

Production of Ectopically Expressing Cell Lines via Lentiviral Transduction

To produce lentiviral particles, 4×10^5 HEK293T cells were seeded on collagen-coated 6-well plates. The following day, the cells were transfected with the plasmids pcz-VSV-G, pCMV-dR8.74 along with plasmids encoding the desired transgenes using Lipofectamine 2000 (Invitrogen, Cat. Nr. 11668019) following the manufacturer's instructions. Six hours post transfection, the medium was changed and lentiviral particles were harvested 48 h post transfection. Supernatants were filtered (Filtropur 0.45, Sarstedt, Cat. Nr. 83.1826) and supplemented with 0.02 M HEPES and 4 µg/mL polybrene and either used directly or stored at -80 °C. For transduction, HEK293T or HepG2/C3A cells were seeded on a 6-well plate and inoculated with 1 mL of lentiviral particles for 6 – 8 h. Selection of the transduced cells was started 48 – 72 h post transduction using 2.5 µg/mL puromycin or 5 µg/mL blasticidin. Transgene expression was validated via immunofluorescence.

HEK293T reporter assay

HEK293T cells stably expressing RfxCas13d-NLS-2A-Puro were seeded in black 96-well Li-Cor microplates at a density of 1.5×10^4 cells per well. The following day, cells were co-transfected with individual crRNA expression plasmids (pUC_hU6-crScaffold_EF1a-FLAG) and HEV reporter constructs using Lipofectamine 2000 (Invitrogen, Cat. No. 11668019) according to the manufacturer's instructions. Four hours post-transfection, the culture medium was replaced with fresh complete medium. After 48 h, cells were fixed with 3% paraformaldehyde for 2 h at room temperature, permeabilized with 0.2% Triton X-100 for 4 min, and blocked with 5% horse serum under gentle agitation. Cells were then incubated overnight at 4 °C with anti-FLAG primary antibody (Sigma, Cat. No. F3165), washed three times with PBS, and incubated for 2 h at room temperature with Alexa Fluor 555-conjugated anti-mouse secondary antibody (Invitrogen, Cat. No. A-31570). Nuclei were counterstained with DAPI, and fluorescence images were acquired using a Keyence fluorescence microscope. Fluorescence images were analyzed using CellProfiler by segmenting nuclei and expanding these masks to define Cas13d- and GFP-positive cells. Integrated fluorescence intensities were quantified, and cells were classified as FLAG- or GFP-positive based on defined intensity thresholds.

In vitro transcription and electroporation

A plasmid containing the cDNA full-length genome of the Kernow-C1/p6 virus isolate[22,23] was utilized for infectious viral particle production, and a plasmid encoding the sequence of the assembly-deficient subgenomic *Gaussia luciferase* reporter replicon (Kernow-C1/p6 strain

with a truncated ORF2 replaced with a *Gaussia luciferase* gene) was used in replication assays. *In vitro* transcription and electroporation into cells was performed as previously described by Todt et al. and Meister et al.[24,25]. In brief, 5 × 10^6 HepG2 or HepG2/C3A cells were transferred in 400 µL of Cytomix containing 2 mM ATP (Cayman Chemical, #14498) and 5 mM glutathione (Sigma Aldrich, #G4251) and mixed with 5 µg *in vitro* transcribed (IVT) RNA. The cells were electroporated using the Gene Pulser System (BioRad) with 975 µF and 270 V for ~ 20 ms and directly transferred into 10 mL cell culture media and plated on a collagen-coated 10 cm plate for virus production, or transferred into 12.1 mL cell culture media and 2 × 10^4cells per well were seeded on a coated 96-well plate for *Gaussia luciferace* assays.

Production of cell culture-derived HEV (HEV_{CC})

Infectious HEV_{CC} particle production was performed as described by Todt et al. and Meister et al.[24,25]. Briefly, HepG2 cells were electroporated with IVT RNA of full-length Kernow-C1/p6 HEV. To obtain extracellular enveloped HEV_{CC} ($eHEV_{CC}$), culture supernatants were collected 7 days post-transfection and stored at 4 °C and used within a week. Intracellular non-enveloped HEV_{CC} ($neHEV_{CC}$) was prepared from cell lysates by trypsinizing cells, neutralizing with DMEM, centrifuging at 200 × g for 5 min, and resuspending in 1.6 mL of medium per transfection. Cells were subjected to three freeze–thaw cycles using liquid nitrogen and ice, followed by centrifugation at 10,000 × g for 10 min. The clarified supernatant was aliquoted and stored at -80 °C. Virus titers were determined by titrating $neHEV_{CC}$ and/or $eHEV_{CC}$ onto HepG2/C3A cells, followed by fixation and ORF2 staining at 7 days post-infection to quantify focus-forming units per mL (FFU/mL)[25].

HEV infection assays

For HEV infection assays, HepG2/C3A (1.5 × 10^4 cells/well) were seeded in 96-well plates and allowed to adhere overnight. The next day, cells were inoculated with HEV (Kernow-C1/p6; MOI 1). Each condition was tested in triplicate. HepG2/C3A cells were incubated for 3 days before fixation and immunofluorescence staining. For lentiviral delivery of crRNAs using the modified SGL40C.EFS.dTomato backbone, HepG2/C3A cells were seeded as described above. The following day, cells were transduced with 25 µL of lentiviral particles (see above for details on lentivirus production). At 24 h post-transduction, the culture medium was replaced. At 48 h post-transduction, cells were infected with HEV (Kernow-C1/p6) at an MOI of 0.1 and incubated for 4 days before fixation and immunofluorescence staining. All conditions were performed in duplicate.

Cell viability assay

HepG2/C3A cells stably expressing Cas13d-NLS or Cas13d-NCS were seeded in 96 well plates (1.5 × 10^4 cells/well) and allowed to adhere overnight. The next day, cells were

inoculated with 25 µL of lentiviral particles. 24 h after transduction, the medium was replaced and cell viability was assessed 48 h post-transduction using an MTT (3-(4,5-dimethylthiazol-2-yl)-2,5-diphenyltetrazolium bromide) assay. In brief, cells were incubated with MTT substrate (0.5 mg/mL; Biomol, #15655) in culture medium at 37 °C with 5% CO_2 for 1–2 h. The medium was then removed, and 50 µL of DMSO was added to each well. Following a 15 min incubation on a rocking shaker, absorbance was measured at 570 nm using a microplate reader (Tecan Group Ltd). Cells treated with 70% (v/v) ethanol for 10 min prior to the MTT assay served as background controls.

Immunofluorescence staining

Cells were fixed with 3% PFA (Roth, 93351) for 10–20 min, followed by three PBS washes. Permeabilization was performed using 0.2% Triton X-100 (Roth, 3051.3) in PBS for 5 min. After three PBS washes, cells were blocked with 5% horse serum (HS) in PBS for at least 1 h at room temperature (RT). Primary antibodies were diluted 1:1,000 in 5% HS and incubated overnight at 4 °C: anti-FLAG® M2 mouse monoclonal antibody (F1804, Merck) and anti-HA rabbit polyclonal antibody (H6908, Sigma-Aldrich). To detect HEV-infected cells, polyclonal HEV genotype 3 capsid (ORF2)-specific rabbit hyperimmune serum (4086 and 2101[26]) was applied at 1:5,000 dilution in 5% HS and incubated overnight at 4 °C. The next day, cells were washed three times and incubated with the appropriate secondary antibodies diluted 1:1,000 in 5% HS: goat anti-rabbit Alexa Fluor® 488 (A11008, Invitrogen) and donkey anti-mouse Alexa Fluor® Plus 555 (A32773, Invitrogen).. After three additional PBS washes, nuclei were stained with 4′,6-diamidino-2-phenylindole (DAPI, Invitrogen, D1306; 1:10,000 in H_2O) and washed three more times before imaging.

Microscopy and image analysis

Fluorescence images were acquired with a wide-field fluorescence microscope (Keyence BZ-X800E) using a 4 × 0.75- numerical aperture (NA) air-objective. DAPI (358 nm), ORF2 (488 nm) and tdTomato signals were acquired sequentially by using the BZ-X Filter DAPI, BZ-X Filter GFP and BZ-X Filter TxRed (560 nm), respectively. Infections were quantified either by counting foci to determine focus-forming units per mL (FFU/mL) or by calculating the percentage of ORF2-positive cells relative to total nuclei or total crRNA positive cells marked by tdTomato expression using CellProfiler[27].

Bioinformatic analysis

To identify potential crRNA binding sites, reverse complements of the input crRNA sequences were locally aligned against a database of 1143 complete HEV genomes (including 751 HEV-3 genomes) retrieved from NCBI. Alignments were performed using blastn (Version 2.12.0+)[28]. Downstream analyses were implemented in Python (Version 3.10.19), with Biopython (Version

1.85) used to construct a neighbor-joining tree from the full genome sequences. Minimal sets of crRNAs covering the broadest range of genotypes or database sequences were derived by ranking and filtering blast hits. Tree visualization was conducted in R (Version 4.5.2) [R Core Team. (2021). R: A language and environment for statistical computing] using ggtree (Version 4.0.4).

Statistical analysis and image processing software

Graphs were plotted and statistical analysis was performed with GraphPad PRISM version 10.2.1 for Windows (https://www.graphpad.com). Statistical significance against non-targeting control (NTCR) determined using a one-way ANOVA with Dunnett's post hoc test (***P < 0.001, **P < 0.01). Images were analysed with FIJI version 2.16.0 (https://imagej.net/software/fiji/) and/or CellProfiler[27]. Final graphics were edited using Adobe Illustrator 2024 (https://www.adobe.com/).

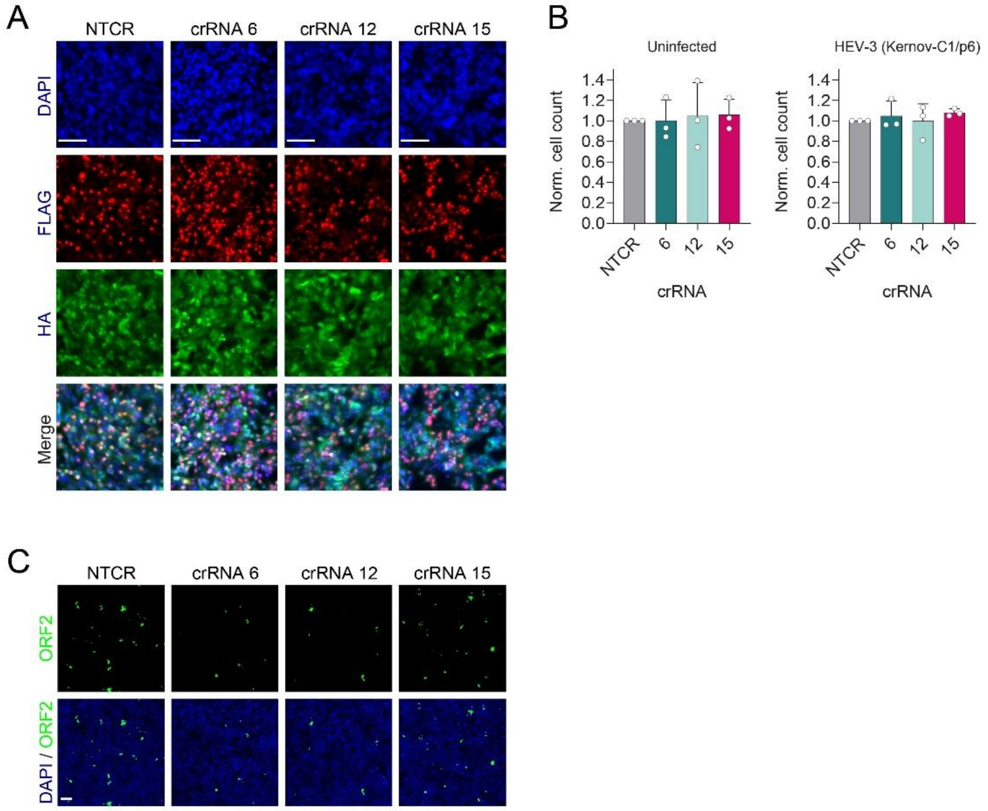

Fig. S1: (A) Representative immunofluorescence images of HepG2/C3A cells stably expressing Cas13d-NLS-FLAG (FLAG staining) and crRNA (HA staining). Nuclei were stained with DAPI (blue). (B) Relative cell number based on nuclear counts of HepG2/C3A cells stably expressing Cas13d and crRNA, with or without 72 h post-infection with HEV (Kernow-C1/p6; MOI 1) (means + SD; n = 3). (C) Representative immunofluorescence images of HepG2/C3A cells expressing Cas13d-NLS and a crRNA stained for viral capsid protein (ORF2) and used for quantification of viral progeny production (Figure 1I, J). Nuclei are stained with DAPI (blue). All scale bars = 100 µm.

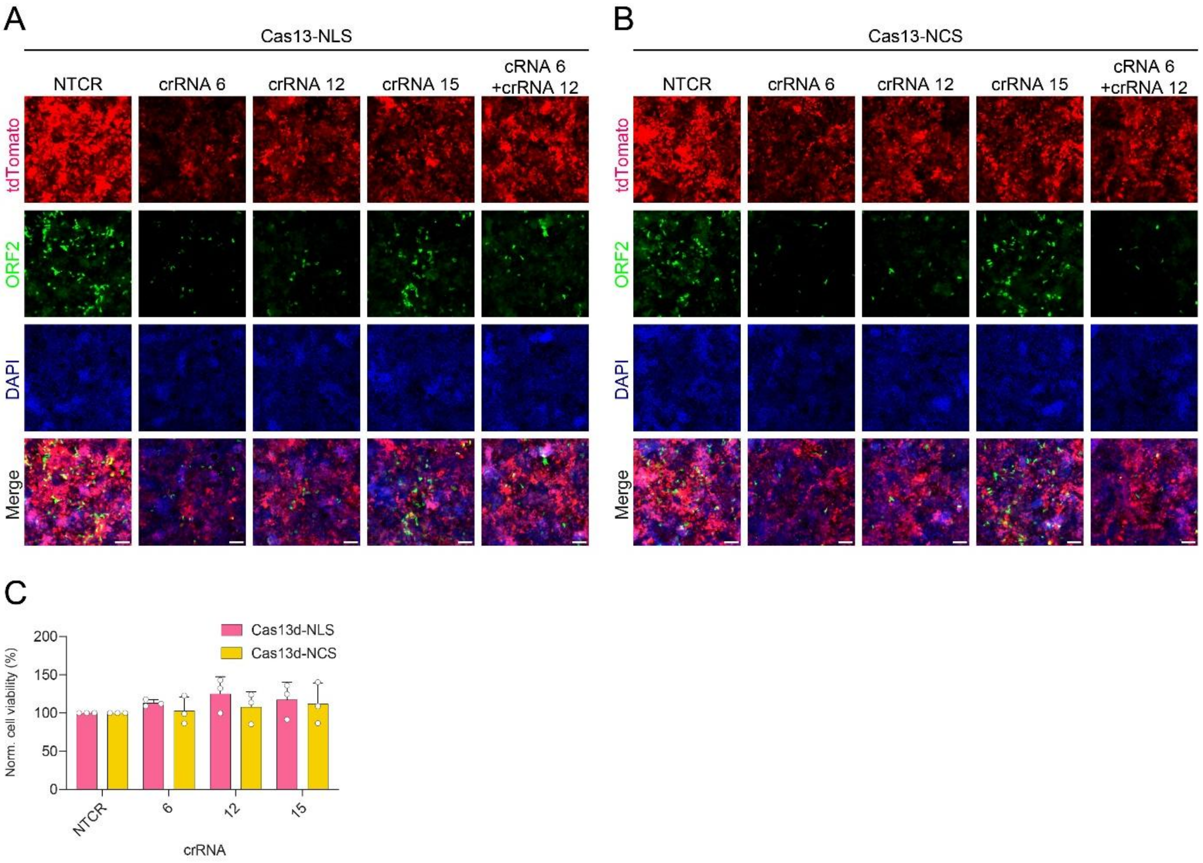

Fig. S2: (A and B) Representative immunofluorescence images of HepG2/C3A cells stably expressing Cas13d-NLS (A) or Cas13d-NCS (B) after crRNA delivery via lentiviral transduction for 48 h, followed by infection with HEV (Kernow-C1/p6; MOI 1) for 96 h. Nuclei were stained with DAPI (blue). All scale bars = 100 µm. (C) Normalized cell viability (%) of HepG2/C3A cells stably expressing Cas13d-NLS-FLAG or Cas13d-NCS-FLAG following crRNA transduction and without 96 h post-infection with HEV (Kernow-C1/p6; MOI 0.1) (means + SD; n = 3).

Table S1: Spacer sequences used in the study.

crRNA	Target	Spacer (5‘-3‘)
NTCR	NTCR	acaaatctatctgaataaactcttcttc
sfGFP	sfGFP	attcaacaagaattgggacaact
1	ORF1.1	ccgaaccaccacagcattcgcca
2	ORF1.1	aaaaccaactgccggggttgcat
3	ORF1.1	gcataaaactggagctggcggcc
4	ORF1.1	aaacatcatggttatagcccgca
5	ORF1.1	cagatagtcagataagccgcagt
6	ORF1.1	aaaacagcagaatttacccgcga
7	ORF1.2	acaacatcaacacagacctgcgc
8	ORF1.2	ccaataaggttatgtaccagccc
9	ORF1.2	gtaaactgatagtgcacaatccc
10	ORF1.2	aattattgacaatcacatccgag
11	ORF1.2	gaaaaatgtgatgcgcgagacat
12	ORF1.2	caaccttcaatttaagcccacag
13	ORF2	gaatcaaccctgtcaccccagaa
14	ORF2	agggatgactaactcggaggcaa
15	ORF2	gtcaccccagaaaccaccgccgg
16	ORF2	cgacgaaatcaattctgtcggta
17	ORF2	gtctcaacagagcgccagccttg
18	ORF2	tcaacatcaggtacaggggctgt
19	ORF2	gaatgcaaagcattaccagaccg
20	ORF2	atagaaatagcataaccaccaac
21	ORF2	agtgcaaaatcaaggagccccaa
22	ORF2	agaaacacgaagaacagcagcag
