# Supplementary material for: Development of a CRISPR-Cas13-based antiviral strategy against hepatitis E virus
Source: JHEP Rep. 2026 May 4;8(7):101885. doi: 10.1016/j.jhepr.2026.101885 (PMC13277442; doi:10.1016/j.jhepr.2026.101885)
Supplement: Multimedia component 2 [file mmc2.pdf]

JOURNAL
OF HEPATOLOGY

Journal of Hepatology

CTAT methods

Tables for a "Complete, Transparent, Accurate and Timely account" (CTAT) are now mandatory for all revised submissions. The aim is to enhance the reproducibility of methods.

- Only include the parts relevant to your study
- Refer to the CTAT in the main text as 'Supplementary CTAT Table'
- Do not add subheadings
- Add as many rows as needed to include all information
- Only include one item per row

If the CTAT form is not relevant to your study, please outline the reasons why:

1.1 Antibodies

Name	Citation	Supplier	Cat no.	Clone no.
Anti-FLAG M2 mouse monoclonal antibody	—	Sigma-Aldrich / Merck	F1804 / F3165	M2
Anti-HA rabbit polyclonal antibody	—	Sigma-Aldrich	H6908	Polyclonal
HEV genotype 3 capsid (ORF2)-specific rabbit hyperimmune serum	—	In-house (hyperimmune serum 4086 and 210126)	4086 / 210126	Polyclonal
Goat anti-rabbit Alexa Fluor 488	—	Invitrogen	A11008	—
Donkey anti-mouse Alexa Fluor Plus 555	—	Invitrogen	A32773	—
Alexa Fluor 555-conjugated anti-mouse secondary antibody	—	Invitrogen	A-31570	—

1.2 Cell lines

Name	Citation	Supplier	Cat no.	Passage no.	Authentication test method
HepG2	—	ATCC	HB-8065		Microsynth Cell Line Authentication Service
HEK293T	—	ATCC	CRL-3216		Microsynth Cell Line

					Authentication Service
HepG2/C3A	—	Kindly provided by Charles Rice (Rockefeller University, USA)	—		Microsynth Cell Line Authentication Service

1.3 Organisms

Name	Citation	Supplier	Strain	Sex	Age	Overall n number

Not applicable — no animal models were used in this study.

1.4 Sequence based reagents

Name	Sequence	Supplier
crRNAs targeting HEV	See Table S1	Custom synthesized oligonucleotides (supplier not specified)
Modified Cas13d direct repeat	Derived from pLentiRNAGuide_001 (Addgene #138150)	Addgene
HEV-ORF1.1 sensor	Derived from Kernow-C1/p6 genome; cloned into pSLQ5079 backbone	Cloned in-house
HEV-ORF1.2 sensor	Derived from Kernow-C1/p6 genome; cloned into pSLQ5079 backbone	Cloned in-house
HEV-ORF2 sensor	Derived from Kernow-C1/p6 genome; cloned into pSLQ5079 backbone	Cloned in-house
Blast-2A-RfxCas13d-NLS	RfxCas13d-2xNLS with blasticidin resistance cassette replacing mCherry	Cloned in-house
Blast-2A-RfxCas13d-NCS	RfxCas13d fused to NLS–NLS–NES (NCS sequence; PMID: 38609360)	Cloned in-house
hU6-crScaffold_EF1a-tdTomato	Cas13d direct repeat inserted into SGL40C.EFS.dTomato backbone	Cloned in-house
RfxCas13d-NLS-2A-Puro	Cas13d-NLS construct derived from pLentiRNACRISPR_005 with hU6-DR_BsmBI deletion	Cloned in-house
pUC_hU6-crScaffold_EF1a-FLAG	3×FLAG inserted replacing BFP in pSLQ5429 backbone	Cloned in-house

1.5 Biological samples

Description	Source	Identifier
Full-length HEV genome plasmid (Kernow-C1/p6)	Gift from Sue Emerson	Kernow-C1/p6
Gaussia luciferase HEV replicon	Gift from Sue Emerson	Kernow-C1/p6 GLuc replicon
Database of 1143 complete HEV genomes	NCBI	1143 genomes (including 751 HEV-3)

1.6 Deposited data

Name of repository	Identifier	Link

1.7 Software

Software name	Manufacturer	Version
GraphPad Prism	GraphPad Software	10.2.1
FIJI	ImageJ	2.16.0
CellProfiler	Broad Institute	
Python	Python Software Foundation	3.10.19
Biopython		1.85
R	R Core Team	4.5.2
ggtree	Bioconductor	4.0.4
blastn	NCBI	2.12.0+
Adobe Illustrator	Adobe	2024

1.8 Other (e.g. drugs, proteins, vectors etc.)

pSLQ5079_pHR_PGK_sfGFP_CoV-F1 (Addgene #155303)		
pSLQ5465_pHR_hU6-crScaffold_EF1a-PuroR-T2A-BFP (Addgene #155307)		
pSLQ5429_pUC_hU6-crScaffold_EF1a-BFP (Addgene #155306)		
pSLQ5428_pHR_EF1a-mCherry-P2A-Rfx_Cas13d-2xNLS-3xFLAG (Addgene #155305)		
pLentiRNACRISPR_005 (Addgene #138147)		
pLentiRNAGuide_001 (Addgene #138150)		
pCMVR8.74 (Addgene #22036)		
pMD2.G (Addgene #12259)		
SGL40C.EFS.dTomato (Addgene #89395)		
Full-length HEV genome (Kernow-C1/p6)	Complete cDNA of HEV genotype 3 strain Kernow-C1/p6	Gift from Sue Emerson

HEV Gaussia luciferase replicon	Kernow-C1/p6 with truncated ORF2 replaced by Gaussia luciferase	Gift from Sue Emerson

1.9 Please provide the details of the corresponding methods author for the manuscript:

**Yannick Brüggemann**

2.0 Please confirm for randomised controlled trials all versions of the clinical protocol are included in the submission. These will be published online as supplementary information.

**Not applicable — this study does not involve a randomized controlled clinical trial.**
