# Supplementary material for: Development of a CRISPR-Cas13-based antiviral strategy against hepatitis E virus
Source: JHEP Rep. 2026 May 4;8(7):101885. doi: 10.1016/j.jhepr.2026.101885 (PMC13277442; doi:10.1016/j.jhepr.2026.101885)
Supplement: Multimedia component 4 [file mmc4.pdf]

Development of a CRISPR-Cas13-based antiviral strategy against hepatitis E virus

Authors

Emely Richter, Mara Klöhn, Maximilian K. Nocke, …, Daniel Todt, Eike Steinmann, Yannick Brüggemann

Correspondence

yannick.brueggemann@ruhr-uni-bochum.de (Y. Brüggemann).

Graphical abstract

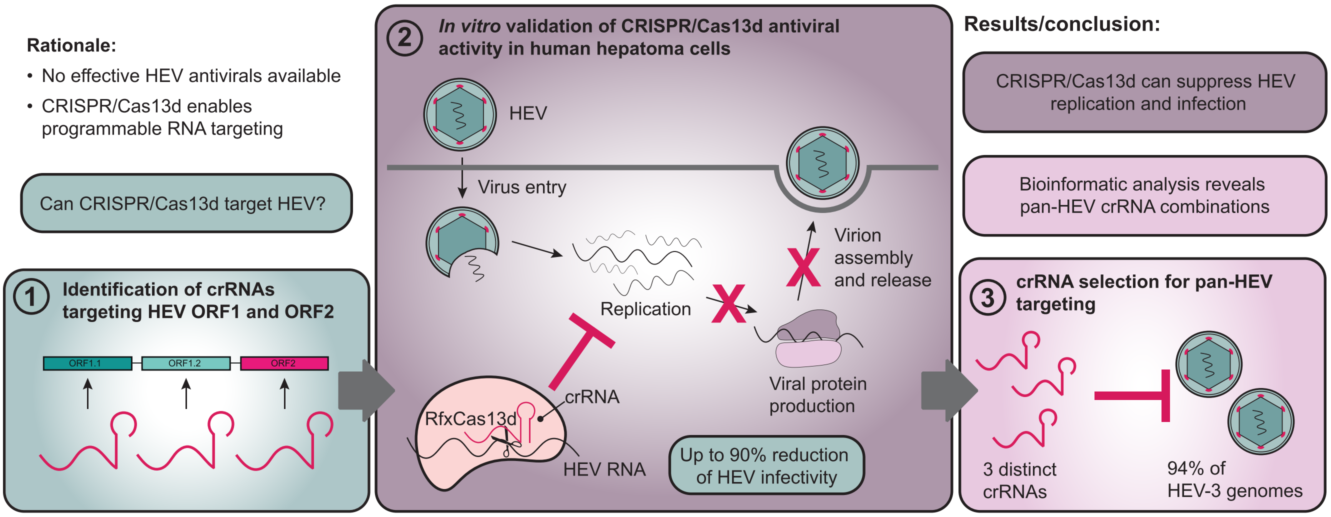

Highlights:

- CRISPR/Cas13d enables efficient targeting of HEV RNA sequences.
- ORF1-targeting crRNAs reduce viral replication, capsid expression, and infectious HEV production in hepatoma cells.
- A combination of three to four crRNAs provides broad coverage of circulating HEV genomes, potentially buffering viral escape mutations.

Impact and implications:

This study establishes CRISPR/Cas13d as a proof-of-concept antiviral strategy against hepatitis E virus (HEV), demonstrating suppression of viral replication and particle production *in vitro*. By identifying a minimal set of broadly effective crRNAs, we provide a framework for targeting diverse HEV variants and buffering against viral evolution. These findings highlight the potential of CRISPR-based systems as innovative antiviral strategies.

https://doi.org/10.1016/j.jhepr.2026.101885
© 2026 The Authors. Published by Elsevier B.V. on behalf of European Association for the Study of the Liver (EASL). This is an open access article under the CC BY license (http://creativecommons.org/licenses/by/4.0/).

JHEP|Reports

Development of a CRISPR-Cas13-based antiviral strategy against hepatitis E virus

Emely Richter[1,2], Mara Klöhn[1,2], Maximilian K. Nocke[1,2,3,4], Marcel Edgar Friedrich[1,2], Daniel Todt[1,2,3,4], Eike Steinmann[1,2,5], Yannick Brüggemann[1,2,*]

JHEP Reports **2026**. vol. 8 | 1–6

Check for updates

Background & Aims: Effective antiviral drugs remain unavailable for many clinically relevant pathogens, including the hepatitis E virus (HEV). This study aimed to evaluate the CRISPR/Cas13d system as a potential antiviral strategy against HEV.

Methods: We developed a reporter assay to screen CRISPR RNAs (crRNAs) targeting conserved regions of the HEV genome and tested their antiviral activity in human hepatoma cells using a robust HEV cell culture model. HEV replication was assessed using a subgenomic replicon, infectious particle production was quantified by immunofluorescence and titration assays. A bioinformatic analysis was performed to identify a minimal set of crRNAs capable of broadly targeting circulating human pathogenic HEV strains.

Results: A crRNA screen identified multiple functional crRNAs targeting HEV-3, with ORF1-targeting crRNAs significantly reducing viral capsid expression (p <0.01) and the number of HEV-infected cells (p <0.01). Cas13d-mediated targeting led to robust reduction of HEV replication and markedly lowered infectious virus production *in vitro* (p <0.001). Bioinformatic analysis revealed that just three distinct crRNAs could cover ~94% of known HEV genomes with zero mismatches, while four crRNAs achieved complete coverage.

Conclusions: Our findings demonstrate that CRISPR/Cas13d can target HEV replication and viral progeny production *in vitro*. The identification of a minimal crRNA set capable of broadly targeting circulating HEV strains suggests that the CRISPR/Cas13d system may offer an antiviral strategy to address challenges related to viral evolution and treatment escape.

© 2026 The Authors. Published by Elsevier B.V. on behalf of European Association for the Study of the Liver (EASL). This is an open access article under the CC BY license (http://creativecommons.org/licenses/by/4.0/).

Introduction

The hepatitis E virus (HEV, species *Paslahepevirus balayani*) is a long-overlooked RNA virus and the primary cause of acute viral hepatitis in humans globally. Each year, HEV causes an estimated 20 million infections, 3.3 to 19 million acute cases, and 3,450 to 70,000 deaths, with wide estimate ranges due to inconsistent surveillance and reporting, especially in low- and middle-income countries.[1,2] In addition, HEV ranks sixth among viruses with a high spillover risk, underscoring its significant potential for zoonotic transmission.[3] While HEV infections are typically self-limiting and asymptomatic in immunocompetent individuals, they can progress to chronicity in immunocompromised patients and cause fulminant hepatitis in high-risk groups, such as pregnant women.[4,5] Current therapeutic options for HEV are limited to the off-label use of the broad-spectrum antiviral agent ribavirin (RBV).[6–8] However, RBV is contraindicated in pregnancy owing to its teratogenicity and its use is further restricted by suboptimal efficacy, poor tolerability, and a range of side effects. Moreover, HEV variants emerging in response to antiviral treatment have been identified.[9–12] Although the emergence of viral variants may contribute to RBV resistance, a causal link to treatment failure has yet to be established. Collectively, these limitations underscore an urgent need for the development of novel and safer antiviral strategies.

In this context, the discovery of RNA-targeting CRISPR/Cas systems in bacteria has generated tremendous interest in antiviral research.[13] The CRISPR/Cas13 system functions similarly to the well known CRISPR/Cas9 system. However, unlike the Cas9 endonuclease which targets DNA, the Cas13 enzyme targets and cleaves single-stranded RNA. The diverse Cas13 family contains at least four known subtypes (Cas13a, Cas13b, Cas13c, and Cas13d). Cas13 enzymes use so-called CRISPR-associated RNAs (crRNAs) that contain a customizable 22-nt spacer sequence that can direct the Cas13 protein to specific RNA molecules for targeted RNA degradation.[14] Hence, the specific RNA endonuclease activity of the Cas13 protein can be "programmed" to specifically recognize and target transcripts or viral RNA genomes. In initial studies, Cas13 was demonstrated to mitigate enterovirus, influenza and SARS-CoV-2 infections *in vitro* in human cells and *in vivo* in rodent models.[15–17] Here we explored whether Cas13d could be used to target HEV *in vitro* in human cells in an analogous manner.

* Corresponding author. Address: Department of Molecular and Medical Virology, Ruhr University Bochum, Bochum, Germany.
E-mail address: yannick.brueggemann@ruhr-uni-bochum.de (Y. Brüggemann).
https://doi.org/10.1016/j.jhepr.2026.101885

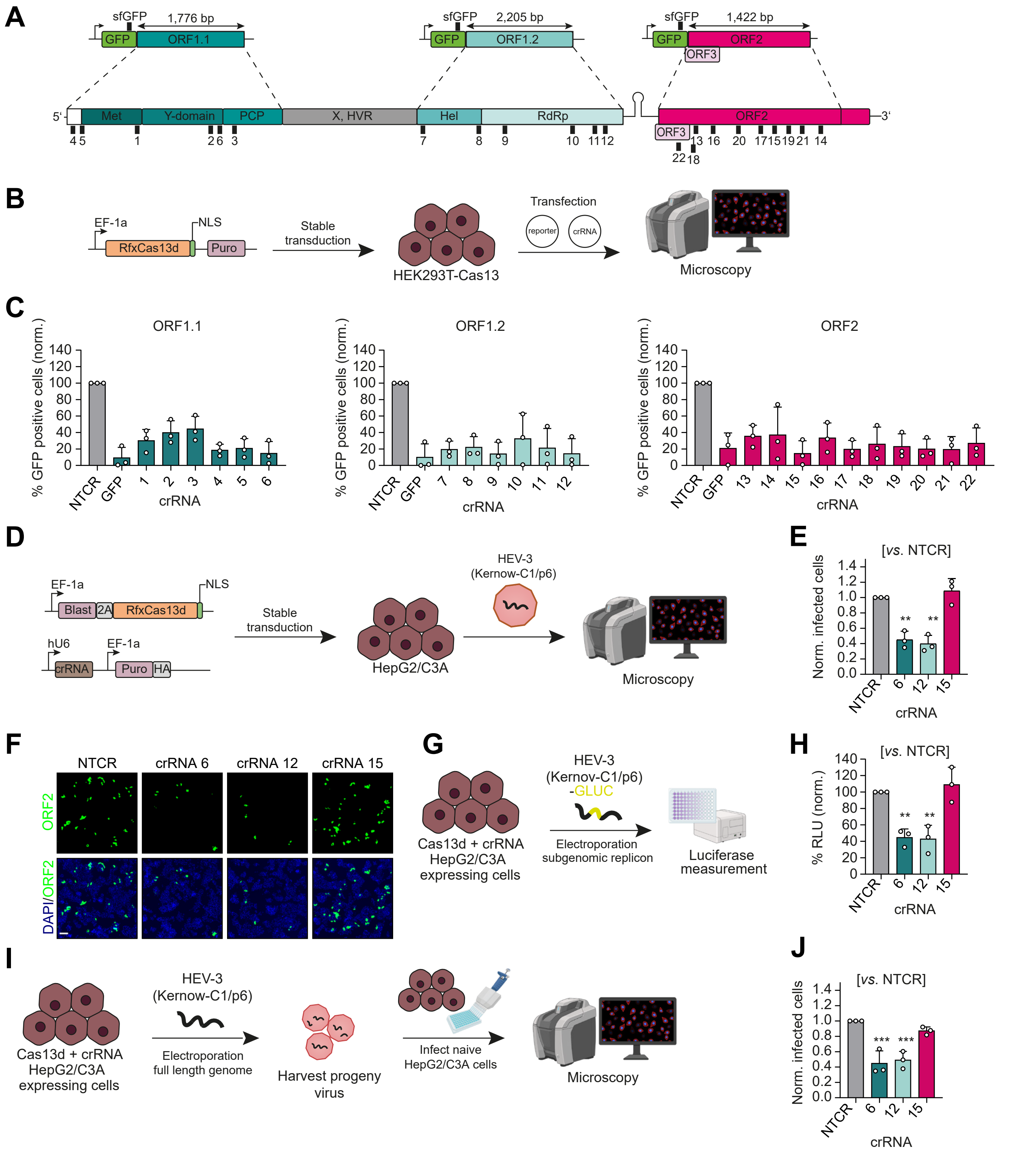

Fig. 1. Cas13d-mediated targeting of HEV *in vitro*. (A) Schematic of HEV reporters (ORF1.1; ORF1.2 and ORF2) created with sequences of the HEV-3/p6 fused to sfGFP and selected crRNAs. (B) Experiment workflow to challenge Cas13d-NLS expressing HEK293T cells with HEV reporters and crRNAs. (C) Quantification of relative GFP expression 24 h after transfection (means + SD; n = 3). ORF1.1 (left); ORF1.2 (middle) and ORF2 (right). (D) HepG2/C3A cells stably expressing Cas13d-NLS-FLAG and a crRNA were infected with HEVp6 and stained for viral capsid protein after 3 days. (E) Quantification of relative infected cells (means + SD; n = 3). (F) Immunofluorescence images of HepG2/C3A cells 72 h after infection with HEV (Kernow-C1/p6; MOI 1). Viral capsid protein (ORF2) = green; DAPI = blue. Scale bar = 100 µm. (G) HepG2/C3A cells stably expressing Cas13d-NLS-FLAG and a crRNA were electroporated with HEV p6 GLUC RNA (Kernow-C1/p6-GLUC). GLUC signal in supernatant was quantified 3 days after electroporation. (H) RLU for each crRNA after HEV p6 GLUC electroporation normalized to the NTCR (means + SD; n = 3). (I) HepG2/C3A stably expressing Cas13d-NLS-FLAG and a crRNA were electroporated with HEV full-length RNA (Kernow-C1/p6). Virus was harvested and titrated after 7 days. The number of infected cells was quantified 7 days after titration. (J) Relative infected cells for each Cas13d/crRNA cell line after virus titration for infectious

Materials and methods

Cell culture

HepG2 (ATCC-Nr.: HB-8065) and HEK293T cells (ATCC-Nr.: CRL-3216) cells were grown in DMEM-high-glucose (Gibco, #11965) supplemented with 10% (v/v) FCS (Capricorn, Lot. Nr. CPC21-4114), 1% (v/v) non-essential amino acids (Gibco, #11140050), 2 mM L-glutamine (Gibco, #25030), 100 IU/ml penicillin and 100 µg/ml streptomycin (Gibco, #15140) (DMEM complete). HepG2/C3A cells (ATCC-Nr.: HB-8065) were grown in Eagle's minimum essential medium (Gibco, #11095) supplemented with 10% (v/v) ultra-low IgG FCS (Gibco, #16250-078), 1% (v/v) non-essential amino acids, 2 mM L-glutamine, 100 µg/ml gentamicin (Gibco, #15710) and 1 mM sodium pyruvate (Gibco, #11360). HepG2 and HepG2/C3A cells were grown on rat collagen-coated (SERVA Electrophoresis, #47256.01) cell culture dishes (Sarstedt) and incubated at 37 °C in a 5% CO_2 incubator.

Additional materials and methods describing assays used in this study are specified in the supplementary information.

Results

To identify effective and specific crRNA sequences to target and cleave HEV RNA within cells, we developed a reporter assay based on fragments of the HEV-3 *Kernow-C1/p6* genome fused to GFP (ORF1.1, ORF1.2 and ORF2). Candidate crRNAs targeting conserved regions of the HEV genome were designed using a Cas13d-specific crRNA prediction algorithm.[18] We selected 22 crRNAs targeting conserved regions within the open reading frame 1 (ORF1) or 2 (ORF2). crRNAs with predicted off-target binding in the human transcriptome, defined as any site with ≤2 mismatches, were excluded (Fig. 1A). To test crRNA activity, we created a HEK293T cell line (HEK293T-Cas13d) stably expressing RfxCas13d-nuclear localization sequence (NLS). Reporter and crRNA expressing plasmids were transfected into HEK293T-Cas13d and GFP fluorescence was determined 24 h post-transfection (Fig. 1B). A crRNA targeting GFP was used as a positive control. We observed that all HEV-specific crRNAs reduced reporter expression compared to a non-targeting control, demonstrating that Cas13d is an effective system for targeted degradation of HEV RNA in human cells (Fig. 1C). From this initial screening we selected the most effective crRNAs (6; 12; 15) for each reporter construct to test the antiviral potential of Cas13d against HEV.

We therefore generated HepG2/C3A cells stably expressing RfxCas13d-NLS (HepG2/C3A-Cas13d) along with individual crRNAs (6; 12; 15) and challenged them with HEVp6 (Figs 1D and S1A). Immunofluorescence analysis performed 72 h post-infection revealed a reduction in viral capsid (ORF2) expression in cells expressing on-target crRNAs against ORF1 (6 and 12) compared to non-targeting controls (Fig. 1E, F). In contrast, crRNA 15 targeting ORF2 had no effect on the number of infected cells. Quantification of infected cells confirmed that crRNAs against ORF1 (6 and 12) significantly decreased the number of HEV-infected cells up to ~50% (Fig. 1E, F). Total cell numbers remained unchanged, indicating that combined Cas13/crRNA expression can reduce HEV infection in hepatoma cells without compromising cell viability, even under activation of Cas13d cleavage activity (Fig. S1B). We next examined whether Cas13d could reduce HEV replication and infectious virus production. HepG2/C3A-Cas13d cells stably expressing crRNAs were electroporated with HEV *Gaussia* luciferase RNA, and viral replication was quantified by *Gaussia* luciferase activity in the supernatant (Fig. 1G). Consistent with the infection data, crRNAs targeting ORF1 (6 and 12) caused a pronounced reduction in luciferase signal compared to non-targeting controls (Fig. 1H). In contrast, the ORF2-targeting crRNA 15 had no effect on replication. To assess the impact on infectious particle production, Cas13d/crRNA-expressing cells were electroporated with full-length HEV RNA, and progeny virus was harvested and titrated after 7 days (Fig. 1I). Immunofluorescence staining against ORF2 revealed a strong reduction in infected cells for lines expressing ORF1-targeting crRNAs 6 and 12, whereas the ORF2-targeting crRNA 15 did not reduce viral progeny production (Fig. S1C). Quantification confirmed that ORF1-targeting crRNAs substantially decreased the production of infectious HEV particles (Fig. 1J). Together, these results demonstrate that Cas13d can reduce HEV replication and the generation of infectious virus *in vitro*. However, the efficiency of the Cas13d-NLS construct in our system was only ~50%, which motivated us to investigate alternative designs. To address this limitation, we explored a nucleocytoplasmic shuttling Cas13d (Cas13d-NCS) construct that fuses nuclear localization and export signals to enhance cytosolic RNA targeting.[19] To assess whether Cas13d-NCS improves antiviral activity against HEV, we generated HepG2/C3A cells stably expressing either Cas13d-NLS or Cas13d-NCS (Fig. 2A), delivered crRNAs via lentiviral transduction, and subsequently infected the cells with HEV (Fig. 2B). Compared to Cas13d-NLS, Cas13d-NCS markedly reduced HEV infectivity by up to ~90% with crRNAs targeting ORF1 (6 and 12), whereas crRNA 15 against ORF2 had no effect (Fig. 2C). Total cell numbers (Fig. 2D) and viability (Fig. S2C) were unaffected, indicating that Cas13d-NCS efficiently suppresses HEV infection without compromising cell health. Combining crRNAs 6 and 12, with half the lentivirus used per crRNA, maintained similar levels of HEV suppression (Fig. 2C), indicating that crRNAs can be effectively combined without loss of activity. Because combining individual crRNAs potentially allows for broad targeting of HEV genotypes, we next asked whether it is possible to design a minimal set of crRNAs that could effectively target the majority of known HEV genomes. Through bioinformatic analysis, we aligned 1,143 full-length HEV genomes against our initial panel of crRNAs (excluding the inefficient crRNA 15) (Fig. 1A) and iteratively refined this set to identify the smallest subset of crRNAs capable of targeting all known HEV genomes with zero mismatches (Fig. 2E, F). We found that just three crRNAs could

particle production assay normalized to the NTCR (means + SD; n = 3). Statistical significance in (E), (H) and (J) was determined using a one-way ANOVA with Dunnett's post hoc test. (***p <0.001 and **p <0.01, non-significant values are not shown). GLUC, *Gaussia luciferase*; HEV, hepatitis E virus; MOI, multiplicity of infection; NLS, nuclear localization signal; NTCR, non-targeting control RNA; ORF, open reading frame; RLU, relative light units; sfGFP, superfolder green fluorescent protein.

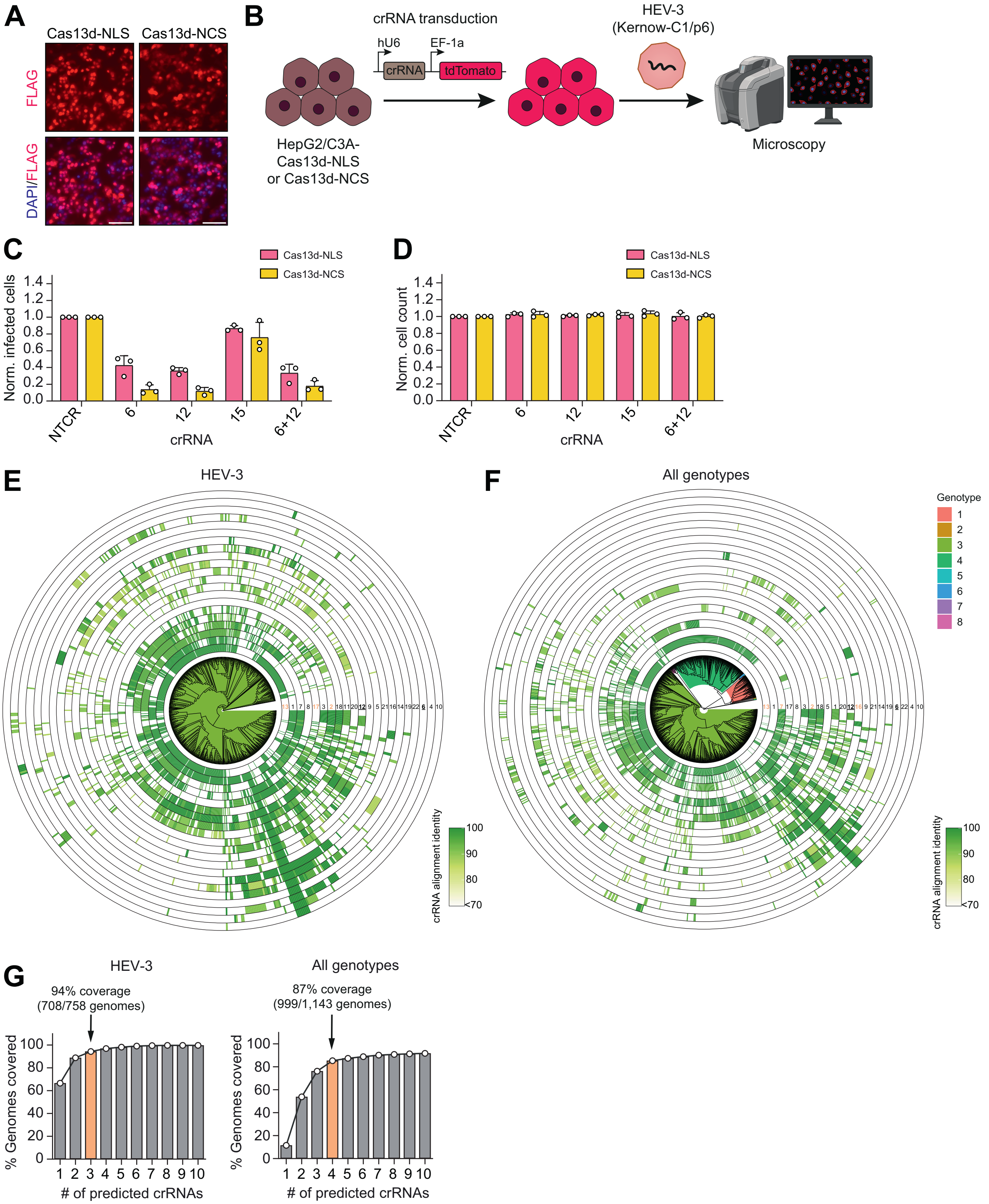

Fig. 2. Cas13d-NCS enhances antiviral activity against HEV and pan-HEV targeting using a minimal crRNA pool. (A) Immunofluorescence images of HepG2/C3A stably expressing Cas13d-NLS-FLAG or Cas13d-NCS-FLAG. Cas13-NLS/NCS-FLAG = red; DAPI = blue. All scale bars = 100 μm. (B) Experiment workflow to challenge Cas13d expressing HEK293T cells with HEV reporters and crRNAs. (C) Quantification of HEV-infected cells and (D) relative cell number based on nuclear counts (D) in HepG2/C3A cells stably expressing Cas13d-NLS-FLAG or Cas13d-NCS-FLAG following crRNA transduction and 96 h post-infection with HEV (Kernow-C1/p6; MOI 0.1). (E and F) Pan-HEV targeting using a minimal crRNA pool. Inner ring: A phylogenetic tree of all (E) 758 HEV-3 genomes or (F) 1,143 genomes representing all HEV genotypes analyzed, organized according to subtype or genotype classification. Outer ring: Coverage of individual HEV genomes by the initially tested 21 complementary crRNAs (excluding crRNA 15), with targeted genomes indicated in green. crRNA 6 and crRNA 12 are highlighted in bold. The minimum set of crRNAs identified in (G) is indicated in orange. (G) Predicted minimum number of crRNAs required to achieve coverage of the majority of analyzed HEV-3 genomes (n = 758) or genomes representing all HEV genotypes (n = 1,143). HEV, hepatitis E virus; MOI, multiplicity of infection; NCS, nucleocytoplasmic shuttling signal; NLS, nuclear localization signal; ORF, open reading frame; NTCR, non-targeting control RNA.

target ~94% (708/758) of HEV-3 genomes, and a panel of four distinct crRNAs covered ~87% (975/1,143) across all HEV genotypes with no mismatches (Fig. 2G). The ability to use a relatively small number of crRNAs to broadly target all HEV strains and thereby also potentially buffer against viral evolution and escape highlights the unique and versatile potential of the CRISPR/Cas13 system as an antiviral strategy.

Discussion

In this study, we repurposed the RNA-guided endonuclease activity of Cas13d to target HEV in human cells. We demonstrated that Cas13d can efficiently degrade HEV RNA, suppress viral replication, and reduce infectious particle production in hepatoma cells. The lack of antiviral activity of the ORF2-targeting crRNA may reflect limited accessibility of the target site. While active in the reporter assay, ORF2 crRNAs failed to suppress HEV replication or particle production, likely because ORF2 is translated from a subgenomic RNA expressed late in infection, rather than the full-length genomic RNA. Testing additional ORF2-targeting crRNAs could clarify whether this limitation is site-specific or reflects broader challenges in targeting the subgenomic RNA. Bioinformatic analysis further revealed that as few as three crRNAs could target ~94% of sequenced HEV-3 genomes, and a panel of four crRNAs could target ~87% (975/1,143) of genomes across all HEV genotypes. Unlike Cas9, Cas13d is capable of processing its own CRISPR array,[20] enabling the encoding of multiple guides within a single construct to simultaneously and effectively suppress diverse viral variants.[21] This ability to achieve broad strain coverage with minimal guides not only buffers against viral evolution and escape but also underscores the unique therapeutic potential of Cas13 compared to conventional antivirals.[22] While our findings establish Cas13d as a proof-of-concept antiviral strategy against HEV *in vitro*, several challenges must be addressed before clinical translation. Importantly, our experiments tested Cas13d as a prophylactic system introduced prior to viral challenge; however, we hypothesize that it could also be effective in reducing viral load post-infection, as previously demonstrated for coronaviruses.[21] In addition, we employed stable Cas13d/crRNA-expressing cell lines, which is not compatible with immediate translational applications. A major barrier to clinical deployment of Cas13 remains the development of safe and efficient *in vivo* delivery methods. Future efforts should integrate crRNA selection strategies with predictive algorithms to streamline guide validation, while optimizing delivery methods in relevant *in vivo* models, determining safe and effective dosing regimens, and assessing the immunogenicity of both Cas13d and its delivery vehicles.[23] Collateral RNA cleavage, reported under specific circumstances, must also be carefully evaluated and minimized by carefully regulating RfxCas13d.[24] As Cas13 biology advances, the discovery of more compact, efficient, and less immunogenic RNA-targeting variants could further enhance its therapeutic potential. Notably, the crRNA discovery and validation pipeline established here for HEV can be readily adapted to other CRISPR systems, such as the recently developed type III CRISPR-Csm platform,[25] offering a generalizable framework for next-generation CRISPR-based HEV antivirals.

Affiliations

[1]*Department of Molecular and Medical Virology, Ruhr University Bochum, Bochum, Germany;* [2]*Hepatitis E Virus Research Hub (HepE-Hub), Bochum, Germany;* [3]*Department of Translational and Computational Infection Research (TRACiR), Ruhr University Bochum, Bochum, Germany;* [4]*European Virus Bioinformatics Center (EVBC), Jena, Germany;* [5]*German Centre for Infection Research (DZIF), External Partner Site, Bochum, Germany*

Abbreviations

crRNA, CRISPR RNAs; HEV, hepatitis E virus; NCS, nucleocytoplasmic shuttling sequence; NLS, nuclear localization sequence; ORF, open reading frame; RBV, ribavirin; NTCR, non-targeting control RNA.

Financial support

ES was supported by the German Research Foundation (STE 1954/16-1) and German Centre for Infection Research (DZIF, TTU 05.823_00). DT was supported by the German Federal Ministry of Research, Technology and Space (BMFTR, project VirBio; 01KI2106). YB was supported by German Research Foundation (DFG, BR 7111/1-1) and the Medical Faculty -FoRUM program of the Ruhr University Bochum (F1030-2021). ER was supported by the Hannover Graduate School for Neurosciences, Infection Medicine and Veterinary Sciences (HGNI) of the University of Veterinary Medicine Hannover (TiHo).

Conflicts of interest

Emely Richter: nothing to disclose. Mara Klöhn: nothing to disclose. Maximilian K. Nocke: nothing to disclose. Daniel Todt: nothing to disclose. Eike Steinmann: nothing to disclose. Yannick Brüggemann: nothing to disclose.

Please refer to the accompanying ICMJE disclosure forms for further details.

Authors' contributions

Contributed to the conception and design of the study: YB, ES. Provided administrative, study supervision, and obtained funding: YB, ES, DT. Performed experiments and substantially contributed to the acquisition of data and its analysis: ER, MKL, MN, MEF, YB. Interpretation of data: ER, MKL, MN, MEF, YB, ES, DT. Drafted the manuscript: YB, ES. Revised the manuscript critically for important intellectual content: All authors.

Data availability

All data supporting the findings of this study are included within the article and its supplementary information. Raw data are available upon reasonable request.

Acknowledgments

We are grateful to Suzanne Emerson for the hepatitis E virus p6 clone. HEV-specific rabbit hyperimmune serum was kindly provided by Rainer Ulrich, Friedrich Loeffler Institute, Germany. We thank all members of the Department for Molecular and Medical Virology, Ruhr-University Bochum, for helpful support, suggestions, and discussions.

Supplementary data

Supplementary data to this article can be found online at https://doi.org/10.1016/j.jhepr.2026.101885.

References

Author names in bold designate shared co-first authorship

[1] **Rein DB**, Stevens GA, Theaker J, et al. The global burden of hepatitis E virus genotypes 1 and 2 in 2005. Hepatol (Baltimore, Md.) 2012;55:988–997. https://doi.org/10.1002/hep.25505.

[2] WHO. Hepatitis E. 2025. Available at: https://www.who.int/news-room/fact-sheets/detail/hepatitis-E.

[3] **Grange ZL**, Goldstein T, Johnson CK, et al. Ranking the risk of animal-to-human spillover for newly discovered viruses. Proc Natl Acad Sci USA 2021;118. https://doi.org/10.1073/pnas.2002324118.

[4] **Wedemeyer H**, Pischke S, Manns MP. Pathogenesis and treatment of hepatitis e virus infection. Gastroenterology 2012;142:1388–1397.e1. https://doi.org/10.1053/j.gastro.2012.02.014.
[5] **Bose PD**, Das BC, Kumar A, et al. High viral load and deregulation of the progesterone receptor signaling pathway: association with hepatitis E-related poor pregnancy outcome. J Hepatol 2011;54:1107–1113. https://doi.org/10.1016/j.jhep.2010.08.037.
[6] **Frericks N**, **Klöhn M**, **Lange F**, et al. Host-targeting antivirals for chronic viral infections of the liver. Antivir Res 2025;234:106062. https://doi.org/10.1016/j.antiviral.2024.106062.
[7] EASL clinical practice guidelines on hepatitis E virus infection. J Hepatol 2018;68:1256–1271. https://doi.org/10.1016/j.jhep.2018.03.005.
[8] **Kamar N**, Izopet J, Tripon S, et al. Ribavirin for chronic hepatitis E virus infection in transplant recipients. The New Engl J Med 2014;370:1111–1120. https://doi.org/10.1056/NEJMoa1215246.
[9] **Debing Y**, **Gisa A**, Dallmeier K, et al. A mutation in the hepatitis E virus RNA polymerase promotes its replication and associates with ribavirin treatment failure in organ transplant recipients. Gastroenterology 2014;147:1008–1011. https://doi.org/10.1053/j.gastro.2014.08.040. e7; quiz e15-6.
[10] **Debing Y**, Moradpour D, Neyts J, et al. Update on hepatitis E virology: implications for clinical practice. J Hepatol 2016;65:200–212. https://doi.org/10.1016/j.jhep.2016.02.045.
[11] **Gömer A**, **Klöhn M**, **Jagst M**, et al. Emergence of resistance-associated variants during sofosbuvir treatment in chronically infected hepatitis E patients. Hepatology 2023;78:1882–1895. https://doi.org/10.1097/HEP.0000000000000514. Baltimore, Md.
[12] **Todt D**, Gisa A, Radonic A, et al. In vivo evidence for ribavirin-induced mutagenesis of the hepatitis E virus genome. Gut 2016;65:1733–1743. https://doi.org/10.1136/gutjnl-2015-311000.
[13] **van Beljouw SPB**, Sanders J, Rodríguez-Molina A, et al. RNA-targeting CRISPR-Cas systems. Nat Rev Microbiol 2022. https://doi.org/10.1038/s41579-022-00793-y.
[14] **Konermann S**, Lotfy P, Brideau NJ, et al. Transcriptome engineering with RNA-targeting type VI-D CRISPR effectors. Cell 2018;173:665–676.e14. https://doi.org/10.1016/j.cell.2018.02.033.
[15] **Blanchard EL**, **Vanover D**, **Bawage SS**, et al. Treatment of influenza and SARS-CoV-2 infections via mRNA-encoded Cas13a in rodents. Nat Biotechnol 2021;39:717–726. https://doi.org/10.1038/s41587-021-00822-w.
[16] **Abbott TR**, **Dhamdhere G**, **Liu Y**, et al. Development of CRISPR as an antiviral strategy to combat SARS-CoV-2 and influenza. Cell 2020;181:865–876.e12. https://doi.org/10.1016/j.cell.2020.04.020.
[17] **Keng CT**, **Yogarajah T**, Lee RCH, et al. AAV-CRISPR-Cas13 eliminates human enterovirus and prevents death of infected mice. EBioMedicine 2023;93:104682. https://doi.org/10.1016/j.ebiom.2023.104682.
[18] **Wessels H-H**, **Méndez-Mancilla A**, Guo X, et al. Massively parallel Cas13 screens reveal principles for guide RNA design. Nat Biotechnol 2020;38:722–727. https://doi.org/10.1038/s41587-020-0456-9.
[19] **Gruber C**, Krautner L, Bergant V, et al. Engineered, nucleocytoplasmic shuttling Cas13d enables highly efficient cytosolic RNA targeting. Cell Discov 2024;10(42). https://doi.org/10.1038/s41421-024-00672-1.
[20] **Yang H**, Patel DJ. Structures, mechanisms and applications of RNA-centric CRISPR-Cas13. Nat Chem Biol 2024;20:673–688. https://doi.org/10.1038/s41589-024-01593-6.
[21] **Zeng L**, **Liu Y**, Wrynla XH, et al. Broad-spectrum CRISPR-mediated inhibition of SARS-CoV-2 variants and endemic coronaviruses in vitro. Nat Commun 2022;13:2766. https://doi.org/10.1038/s41467-022-30546-7.
[22] **Lin X**, **Liu Y**, **Chemparathy A**, et al. A comprehensive analysis and resource to use CRISPR-Cas13 for broad-spectrum targeting of RNA viruses. Cell Rep Med 2021;2:100245. https://doi.org/10.1016/j.xcrm.2021.100245.
[23] **Sharma S**, Myhrvold C. Optimizing the Cas13 antiviral train: cargo and delivery. EMBO Mol Med 2023;15:e17146. https://doi.org/10.15252/emmm.202217146.
[24] **Hart SK**, Müller S, Wessels H-H, et al. Precise RNA targeting with CRISPR-Cas13d. Nat Biotechnol 2025. https://doi.org/10.1038/s41587-025-02558-3.
[25] **Colognori D**, Trinidad M, Doudna JA. Precise transcript targeting by CRISPR-Csm complexes. Nat Biotechnol 2023;41:1256–1264. https://doi.org/10.1038/s41587-022-01649-9.

Keywords: Hepatitis E virus (HEV); CRISPR-Cas13; Antivirals.
Received 24 October 2025; received in revised form 21 April 2026; accepted 26 April 2026; Available online 4 May 2026

Journal of Hepatology, Volume *8*

Supplemental information

Development of a CRISPR-Cas13-based antiviral strategy against hepatitis E virus

Emely Richter, Mara Klöhn, Maximilian K. Nocke, Marcel Edgar Friedrich, Daniel Todt, Eike Steinmann, and Yannick Brüggemann

Development of a CRISPR-Cas13-based antiviral strategy against hepatitis E virus

Emely Richter, Mara Klöhn, Maximilian K. Nocke, Marcel Edgar Friedrich, Daniel Todt, Eike Steinmann, Yannick Brüggemann

Table of contents

Supplementary materials and methods……………………………………………….2

Fig. S1…………………………………………………………………………………….7

Fig. S2…………………………………………………………………………………….8

Table S1…………………………………………………………………………………..9

Supplementary materials and methods

Plasmids

pSLQ5079_pHR_PGK_sfGFP_CoV-F1 (Addgene plasmid #155303), pSLQ5465_pHR_hU6-crScaffold_EF1a-PuroR-T2A-BFP (Addgene plasmid #155307), pSLQ5429_pUC_hU6-crScaffold_EF1a-BFP (Addgene Plasmid #155306) and pSLQ5428_pHR_EF1a-mCherry-P2A-Rfx_Cas13d-2xNLS-3xFLAG (Addgene plasmid #155305) were a gift from Stanley Qi. pLentiRNACRISPR_005 - hU6-DR_BsmBI-EFS-RfxCas13d-NLS-2A-Puro-WPRE (Addgene plasmid plasmid #138147) was a gift from Neville Sanjana. pCMVR8.74 (Addgene plasmid # 22036) and pMD2.G (Addgene plasmid # 12259) were a gift from Didier Trono. SGL40C.EFS.dTomato (Addgene plasmid #89395) was a gift from Dirk Heckl. Plasmids encoding the full-length viral genome of the Kernow-C1/p6 virus isolate or Gaussia luciferase reporter replicon (Kernow-C1/p6 strain with a truncated ORF2 replaced with a Gaussia luciferase gene) were a gift from Sue Emerson. Plasmids were assembled using Gibson assembly (New England Biolabs, E2611L) with inserts PCR-amplified using Q5 high-fidelity DNA polymerase (NEB, M0491L) and primers containing appropriate overhangs. To generate Blast-2A-RfxCas13d-NLS, the mCherry sequence in pSLQ5428_pHR_EF1a-mCherry-P2A-Rfx_Cas13d-2xNLS-3xFLAG (Addgene plasmid #155305) was replaced with a blasticidin resistance gene. Blast-2A-RfxCas13d-NCS was generated by replacing the NLS sequence with a previously described NCS sequence consisting of NLS–NLS–NES. To generate hU6-crScaffold_EF1a-tdTomato, the Cas9 direct repeat in SGL40C.EFS.dTomato was replaced with a modified Cas13d direct repeat from pLentiRNAGuide_001 (Addgene plasmid #138150). To generate RfxCas13d-NLS-2A-Puro, the hU6-DR_BsmBI region of pLentiRNACRISPR_005 was deleted using the Q5 Site-Directed Mutagenesis Kit (NEB, E0554S). To generate pUC_hU6-crScaffold_EF1a-FLAG, the BFP region in pSLQ5429_pUC_hU6-crScaffold_EF1a-BFP was replaced with a 3x FLAG using the Q5 Site-Directed Mutagenesis Kit (NEB, E0554S). HEV sensor sequences, derived from the Kernow-C1/p6 strain, were cloned into pSLQ5079_pHR_PGK_sfGFP_CoV-F1 (Addgene plasmid #155303) by replacing the CoV sequences to generate HEV-ORF1.1, HEV-ORF1.2 or HEV-ORF2 sensors.

crRNA design and cloning

crRNA sequences were designed using the Cas13 design tool (https://cas13design.nygenome.org/. Only crRNAs predicted to have no off-target binding (over 2 mismatches per crRNA) within the human transcriptome were used. crRNA expression plasmids were generated using standard restriction–ligation cloning. In brief, forward and reverse oligonucleotides corresponding to each spacer were phosphorylated, annealed, and ligated into the backbone using T4 DNA ligase. The assembled inserts were cloned into either the pHR backbone (Addgene plasmid #155307), the modified SGL40C.EFS.dTomato

backbone or the pUC19 backbone (Addgene plasmid #155306). All spacer sequences for the crRNAs are listed in Table S1.

Production of Ectopically Expressing Cell Lines via Lentiviral Transduction

To produce lentiviral particles, 4×10^5 HEK293T cells were seeded on collagen-coated 6-well plates. The following day, the cells were transfected with the plasmids pcz-VSV-G, pCMV-dR8.74 along with plasmids encoding the desired transgenes using Lipofectamine 2000 (Invitrogen, Cat. Nr. 11668019) following the manufacturer's instructions. Six hours post transfection, the medium was changed and lentiviral particles were harvested 48 h post transfection. Supernatants were filtered (Filtropur 0.45, Sarstedt, Cat. Nr. 83.1826) and supplemented with 0.02 M HEPES and 4 µg/mL polybrene and either used directly or stored at -80 °C. For transduction, HEK293T or HepG2/C3A cells were seeded on a 6-well plate and inoculated with 1 mL of lentiviral particles for 6 – 8 h. Selection of the transduced cells was started 48 – 72 h post transduction using 2.5 µg/mL puromycin or 5 µg/mL blasticidin. Transgene expression was validated via immunofluorescence.

HEK293T reporter assay

HEK293T cells stably expressing RfxCas13d-NLS-2A-Puro were seeded in black 96-well Li-Cor microplates at a density of 1.5×10^4 cells per well. The following day, cells were co-transfected with individual crRNA expression plasmids (pUC_hU6-crScaffold_EF1a-FLAG) and HEV reporter constructs using Lipofectamine 2000 (Invitrogen, Cat. No. 11668019) according to the manufacturer's instructions. Four hours post-transfection, the culture medium was replaced with fresh complete medium. After 48 h, cells were fixed with 3% paraformaldehyde for 2 h at room temperature, permeabilized with 0.2% Triton X-100 for 4 min, and blocked with 5% horse serum under gentle agitation. Cells were then incubated overnight at 4 °C with anti-FLAG primary antibody (Sigma, Cat. No. F3165), washed three times with PBS, and incubated for 2 h at room temperature with Alexa Fluor 555-conjugated anti-mouse secondary antibody (Invitrogen, Cat. No. A-31570). Nuclei were counterstained with DAPI, and fluorescence images were acquired using a Keyence fluorescence microscope. Fluorescence images were analyzed using CellProfiler by segmenting nuclei and expanding these masks to define Cas13d- and GFP-positive cells. Integrated fluorescence intensities were quantified, and cells were classified as FLAG- or GFP-positive based on defined intensity thresholds.

In vitro transcription and electroporation

A plasmid containing the cDNA full-length genome of the Kernow-C1/p6 virus isolate[22,23] was utilized for infectious viral particle production, and a plasmid encoding the sequence of the assembly-deficient subgenomic *Gaussia luciferase* reporter replicon (Kernow-C1/p6 strain

with a truncated ORF2 replaced with a *Gaussia luciferase* gene) was used in replication assays. *In vitro* transcription and electroporation into cells was performed as previously described by Todt et al. and Meister et al.[24,25]. In brief, 5 × 10^6 HepG2 or HepG2/C3A cells were transferred in 400 µL of Cytomix containing 2 mM ATP (Cayman Chemical, #14498) and 5 mM glutathione (Sigma Aldrich, #G4251) and mixed with 5 µg *in vitro* transcribed (IVT) RNA. The cells were electroporated using the Gene Pulser System (BioRad) with 975 µF and 270 V for ~ 20 ms and directly transferred into 10 mL cell culture media and plated on a collagen-coated 10 cm plate for virus production, or transferred into 12.1 mL cell culture media and 2 × 10^4cells per well were seeded on a coated 96-well plate for *Gaussia luciferace* assays.

Production of cell culture-derived HEV (HEV_{CC})

Infectious HEV_{CC} particle production was performed as described by Todt et al. and Meister et al.[24,25]. Briefly, HepG2 cells were electroporated with IVT RNA of full-length Kernow-C1/p6 HEV. To obtain extracellular enveloped HEV_{CC} ($eHEV_{CC}$), culture supernatants were collected 7 days post-transfection and stored at 4 °C and used within a week. Intracellular non-enveloped HEV_{CC} ($neHEV_{CC}$) was prepared from cell lysates by trypsinizing cells, neutralizing with DMEM, centrifuging at 200 × g for 5 min, and resuspending in 1.6 mL of medium per transfection. Cells were subjected to three freeze–thaw cycles using liquid nitrogen and ice, followed by centrifugation at 10,000 × g for 10 min. The clarified supernatant was aliquoted and stored at -80 °C. Virus titers were determined by titrating $neHEV_{CC}$ and/or $eHEV_{CC}$ onto HepG2/C3A cells, followed by fixation and ORF2 staining at 7 days post-infection to quantify focus-forming units per mL (FFU/mL)[25].

HEV infection assays

For HEV infection assays, HepG2/C3A (1.5 × 10^4 cells/well) were seeded in 96-well plates and allowed to adhere overnight. The next day, cells were inoculated with HEV (Kernow-C1/p6; MOI 1). Each condition was tested in triplicate. HepG2/C3A cells were incubated for 3 days before fixation and immunofluorescence staining. For lentiviral delivery of crRNAs using the modified SGL40C.EFS.dTomato backbone, HepG2/C3A cells were seeded as described above. The following day, cells were transduced with 25 µL of lentiviral particles (see above for details on lentivirus production). At 24 h post-transduction, the culture medium was replaced. At 48 h post-transduction, cells were infected with HEV (Kernow-C1/p6) at an MOI of 0.1 and incubated for 4 days before fixation and immunofluorescence staining. All conditions were performed in duplicate.

Cell viability assay

HepG2/C3A cells stably expressing Cas13d-NLS or Cas13d-NCS were seeded in 96 well plates (1.5 × 10^4 cells/well) and allowed to adhere overnight. The next day, cells were

inoculated with 25 µL of lentiviral particles. 24 h after transduction, the medium was replaced and cell viability was assessed 48 h post-transduction using an MTT (3-(4,5-dimethylthiazol-2-yl)-2,5-diphenyltetrazolium bromide) assay. In brief, cells were incubated with MTT substrate (0.5 mg/mL; Biomol, #15655) in culture medium at 37 °C with 5% CO_2 for 1–2 h. The medium was then removed, and 50 µL of DMSO was added to each well. Following a 15 min incubation on a rocking shaker, absorbance was measured at 570 nm using a microplate reader (Tecan Group Ltd). Cells treated with 70% (v/v) ethanol for 10 min prior to the MTT assay served as background controls.

Immunofluorescence staining

Cells were fixed with 3% PFA (Roth, 93351) for 10–20 min, followed by three PBS washes. Permeabilization was performed using 0.2% Triton X-100 (Roth, 3051.3) in PBS for 5 min. After three PBS washes, cells were blocked with 5% horse serum (HS) in PBS for at least 1 h at room temperature (RT). Primary antibodies were diluted 1:1,000 in 5% HS and incubated overnight at 4 °C: anti-FLAG® M2 mouse monoclonal antibody (F1804, Merck) and anti-HA rabbit polyclonal antibody (H6908, Sigma-Aldrich). To detect HEV-infected cells, polyclonal HEV genotype 3 capsid (ORF2)-specific rabbit hyperimmune serum (4086 and 2101[26]) was applied at 1:5,000 dilution in 5% HS and incubated overnight at 4 °C. The next day, cells were washed three times and incubated with the appropriate secondary antibodies diluted 1:1,000 in 5% HS: goat anti-rabbit Alexa Fluor® 488 (A11008, Invitrogen) and donkey anti-mouse Alexa Fluor® Plus 555 (A32773, Invitrogen).. After three additional PBS washes, nuclei were stained with 4′,6-diamidino-2-phenylindole (DAPI, Invitrogen, D1306; 1:10,000 in H_2O) and washed three more times before imaging.

Microscopy and image analysis

Fluorescence images were acquired with a wide-field fluorescence microscope (Keyence BZ-X800E) using a 4 × 0.75- numerical aperture (NA) air-objective. DAPI (358 nm), ORF2 (488 nm) and tdTomato signals were acquired sequentially by using the BZ-X Filter DAPI, BZ-X Filter GFP and BZ-X Filter TxRed (560 nm), respectively. Infections were quantified either by counting foci to determine focus-forming units per mL (FFU/mL) or by calculating the percentage of ORF2-positive cells relative to total nuclei or total crRNA positive cells marked by tdTomato expression using CellProfiler[27].

Bioinformatic analysis

To identify potential crRNA binding sites, reverse complements of the input crRNA sequences were locally aligned against a database of 1143 complete HEV genomes (including 751 HEV-3 genomes) retrieved from NCBI. Alignments were performed using blastn (Version 2.12.0+)[28]. Downstream analyses were implemented in Python (Version 3.10.19), with Biopython (Version

1.85) used to construct a neighbor-joining tree from the full genome sequences. Minimal sets of crRNAs covering the broadest range of genotypes or database sequences were derived by ranking and filtering blast hits. Tree visualization was conducted in R (Version 4.5.2) [R Core Team. (2021). R: A language and environment for statistical computing] using ggtree (Version 4.0.4).

Statistical analysis and image processing software

Graphs were plotted and statistical analysis was performed with GraphPad PRISM version 10.2.1 for Windows (https://www.graphpad.com). Statistical significance against non-targeting control (NTCR) determined using a one-way ANOVA with Dunnett's post hoc test (***P < 0.001, **P < 0.01). Images were analysed with FIJI version 2.16.0 (https://imagej.net/software/fiji/) and/or CellProfiler[27]. Final graphics were edited using Adobe Illustrator 2024 (https://www.adobe.com/).

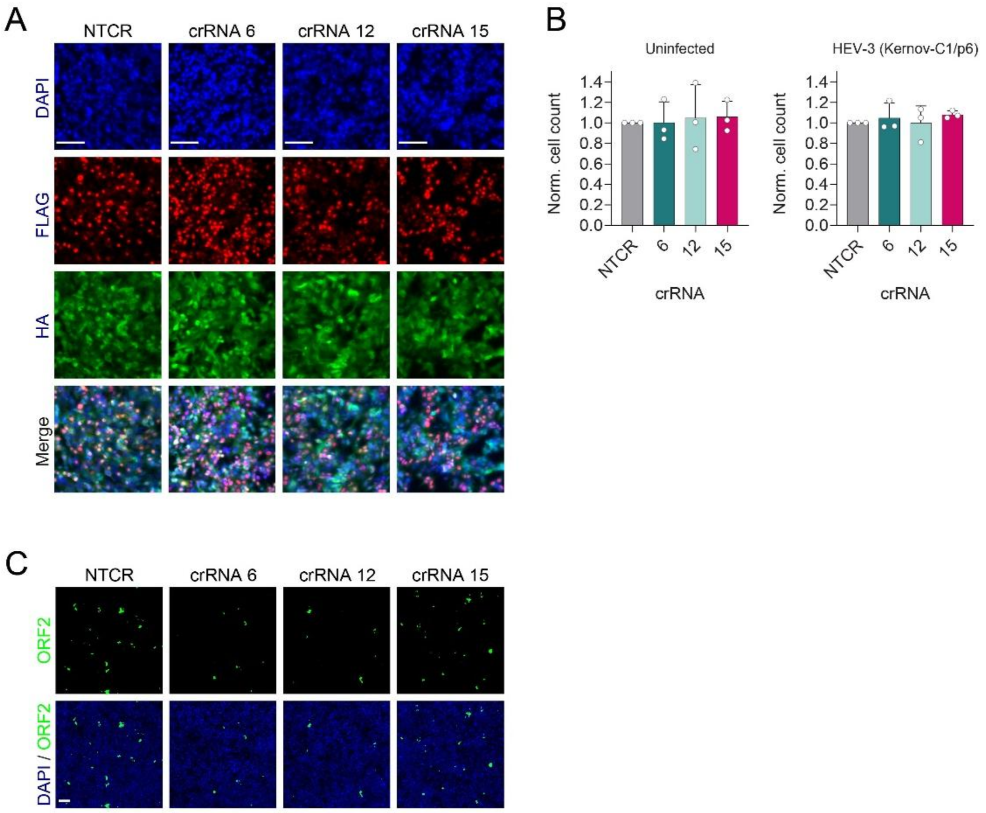

Fig. S1: (A) Representative immunofluorescence images of HepG2/C3A cells stably expressing Cas13d-NLS-FLAG (FLAG staining) and crRNA (HA staining). Nuclei were stained with DAPI (blue). (B) Relative cell number based on nuclear counts of HepG2/C3A cells stably expressing Cas13d and crRNA, with or without 72 h post-infection with HEV (Kernow-C1/p6; MOI 1) (means + SD; n = 3). (C) Representative immunofluorescence images of HepG2/C3A cells expressing Cas13d-NLS and a crRNA stained for viral capsid protein (ORF2) and used for quantification of viral progeny production (Figure 1I, J). Nuclei are stained with DAPI (blue). All scale bars = 100 µm.

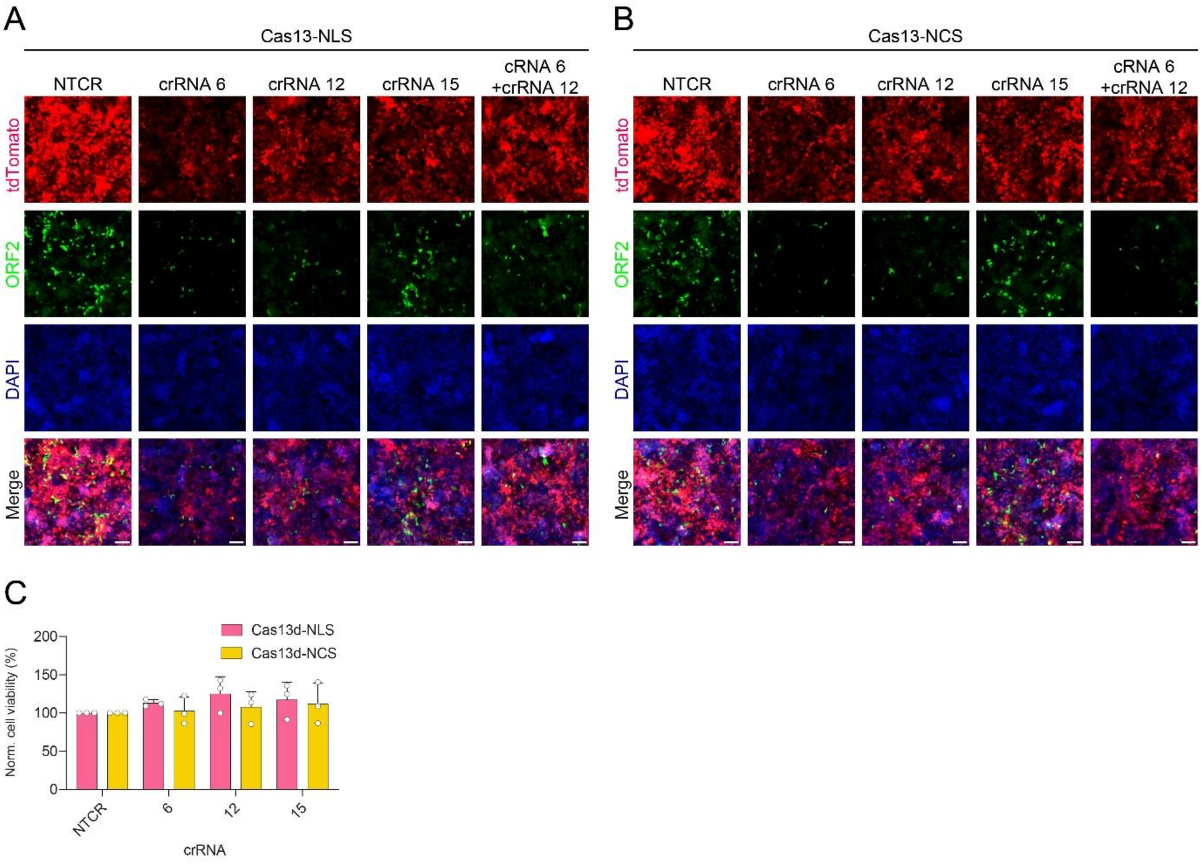

Fig. S2: (A and B) Representative immunofluorescence images of HepG2/C3A cells stably expressing Cas13d-NLS (A) or Cas13d-NCS (B) after crRNA delivery via lentiviral transduction for 48 h, followed by infection with HEV (Kernow-C1/p6; MOI 1) for 96 h. Nuclei were stained with DAPI (blue). All scale bars = 100 µm. (C) Normalized cell viability (%) of HepG2/C3A cells stably expressing Cas13d-NLS-FLAG or Cas13d-NCS-FLAG following crRNA transduction and without 96 h post-infection with HEV (Kernow-C1/p6; MOI 0.1) (means + SD; n = 3).

Table S1: Spacer sequences used in the study.

crRNA	Target	Spacer (5‘-3‘)
NTCR	NTCR	acaaatctatctgaataaactcttcttc
sfGFP	sfGFP	attcaacaagaattgggacaact
1	ORF1.1	ccgaaccaccacagcattcgcca
2	ORF1.1	aaaaccaactgccggggttgcat
3	ORF1.1	gcataaaactggagctggcggcc
4	ORF1.1	aaacatcatggttatagcccgca
5	ORF1.1	cagatagtcagataagccgcagt
6	ORF1.1	aaaacagcagaatttacccgcga
7	ORF1.2	acaacatcaacacagacctgcgc
8	ORF1.2	ccaataaggttatgtaccagccc
9	ORF1.2	gtaaactgatagtgcacaatccc
10	ORF1.2	aattattgacaatcacatccgag
11	ORF1.2	gaaaaatgtgatgcgcgagacat
12	ORF1.2	caaccttcaatttaagcccacag
13	ORF2	gaatcaaccctgtcaccccagaa
14	ORF2	agggatgactaactcggaggcaa
15	ORF2	gtcaccccagaaaccaccgccgg
16	ORF2	cgacgaaatcaattctgtcggta
17	ORF2	gtctcaacagagcgccagccttg
18	ORF2	tcaacatcaggtacaggggctgt
19	ORF2	gaatgcaaagcattaccagaccg
20	ORF2	atagaaatagcataaccaccaac
21	ORF2	agtgcaaaatcaaggagccccaa
22	ORF2	agaaacacgaagaacagcagcag
